# UTP11 deficiency suppresses cancer development via nucleolar stress and ferroptosis

**DOI:** 10.1016/j.redox.2023.102705

**Published:** 2023-04-17

**Authors:** Yu Gan, Jun Deng, Qian Hao, Yingdan Huang, Tao Han, Jin-Guo Xu, Min Zhao, Litong Yao, Yingying Xu, Jianping Xiong, Hua Lu, Chunmeng Wang, Jiaxiang Chen, Xiang Zhou

**Affiliations:** a Department of Physiology, School of Basic Medical Sciences, Nanchang University, Nanchang, 330006, China; b Fudan University Shanghai Cancer Center and Institutes of Biomedical Sciences, Fudan University, Shanghai, 200032, China; c Department of Oncology, The First Affiliated Hospital of Nanchang University, Nanchang, 330006, China; d Department of Oncology, Shanghai Medical College, Fudan University, Shanghai, 200032, China; e Institutes of Health Central Plains, Xinxiang Medical University, Xinxiang, 453003, China; f School of Science, Technology and Engineering, University of the Sunshine Coast, Maroochydore DC, Queensland, 4558, Australia; g Department of Breast Surgery, The First Affiliated Hospital of China Medical University, Shenyang, 110001, China; h Department of Biochemistry & Molecular Biology and Tulane Cancer Center, Tulane University School of Medicine, New Orleans, LA, 70112, USA; i Department of Musculoskeletal Oncology, Fudan University Shanghai Cancer Center, Shanghai, 200032, China; j Key Laboratory of Breast Cancer in Shanghai, Fudan University Shanghai Cancer Center, Fudan University, Shanghai, 200032, China; k Shanghai Key Laboratory of Medical Epigenetics, International Co-laboratory of Medical Epigenetics and Metabolism (Ministry of Science and Technology), Institutes of Biomedical Sciences, Fudan University, Shanghai, 200032, China

**Keywords:** UTP11, Ribosome biogenesis, Nucleolar stress, p53, SLC7A11, Ferroptosis

## Abstract

The eukaryotic ribosome is essential for cancer cell survival. Perturbation of ribosome biogenesis induces nucleolar stress or ribosomal stress, which restrains cancer growth, as rapidly proliferating cancer cells need more active ribosome biogenesis. In this study, we found that UTP11 plays an important role in the biosynthesis of 18S ribosomal RNAs (rRNA) by binding to the pre-rRNA processing factor, MPP10. UTP11 is overexpressed in human cancers and associated with poor prognoses. Interestingly, depletion of UTP11 inhibits cancer cell growth *in vitro* and *in vivo* through p53-depedednt and -independent mechanisms, whereas UTP11 overexpression promotes cancer cell growth and progression. On the one hand, the ablation of UTP11 impedes 18S rRNA biosynthesis to trigger nucleolar stress, thereby preventing MDM2-mediated p53 ubiquitination and degradation through ribosomal proteins, RPL5 and RPL11. On the other hand, UTP11 deficiency represses the expression of SLC7A11 by promoting the decay of NRF2 mRNA, resulting in reduced levels of glutathione (GSH) and enhanced ferroptosis. Altogether, our study uncovers a critical role for UTP11 in maintaining cancer cell survival and growth, as depleting UTP11 leads to p53-dependent cancer cell growth arrest and p53-independent ferroptosis.

## 1 Introduction

Robust ribosome biogenesis is vital for maintaining an active translational machinery that is needed for protein production in rapidly growing cancer cells [1]. This process involves approximately 70 ribosomal proteins (RPs), four ribosomal RNA (rRNA) species, including 28S, 18S, 5.8S, and 5S rRNAs, as well as over 150 auxiliary factors [2]. It is finely coordinated with cell growth and proliferation and highly regulated by oncogenic proteins and tumor suppressors [2]. For instance, oncogenic c-MYC promotes ribosome biogenesis and global protein synthesis by increasing the expression of rRNAs and RPs [3], whereas the tumor suppressor p53 represses RNA Pol I- and III-mediated transcription, resulting in the reduction in rRNA and ribosome biogenesis [[4], [5], [6]].

Ribosomal or nucleolar stress often occurs when any step of the ribosome biogenesis is disrupted by genetic alterations, nutrient depletions, or therapeutic agents, consequently leading to cell growth arrest and apoptosis [2,7,8]. Earlier studies showed that several RPs, such as RPL5, RPL11, and RPL23, are involved in this process [[9], [10], [11], [12], [13]]. Specifically, when cells are grown under nucleolar stress, several RPs with RPL5 and RPL11 as key players are released from the nucleolus to the nucleoplasm where they bind to MDM2 or p53, preventing MDM2-mediated p53 degradation and consequent and activation [14,15]. This is supported by numerous studies showing that deficiencies of the ribosome biogenesis-associated factors, such as RPS14, RPS19, SPIN1, and SBDS [[16], [17], [18]], and the nucleolar stress-inducing agents, such as 5-fluorouracil, mycophenolic acid, inauhzin, and olaparib [[19], [20], [21], [22]], induce p53 stabilization and activation in an RPL5/RPL11 dependent manner. In addition, later studies revealed that ribosome-free RPs can also regulate cancer cell growth, migration, and autophagy through c-MYC, JAK/STAT3, and mTOR signaling pathways in response to nucleolar stress [23]. Thus, the extra-ribosome functions of RPs can be exerted in both p53-dependent and independent fashions.

Once activated, p53 promotes ferroptosis in addition to cell cycle arrest and apoptosis. Ferroptosis is an iron-dependent regulated cell death [24,25] and initiated by peroxidation of polyunsaturated fatty acid-containing phospholipid, which is catalyzed by ferrous iron or lipoxygenases. The system x_c_-cystine/glutamate antiporter, which is composed of SLC7A11 and SLC3A2, is critical for the uptake of cystine and biosynthesis of glutathione (GSH), a potent reductant that detoxifies lipid peroxides and antagonizes ferroptosis [26]. Interestingly, p53 can either protect cells from oxidative damage by upregulating SLC7A11 [27] or promote ferroptosis by downregulating SLC7A11 [24]. The expression of SLC7A11 is also upregulated by two oncogenic transcription factors, NRF2 and ATF4, thus suppressing ferroptosis and consequently enhancing cancer cell survival [28,29]. Targeting SLC7A11 has been explored to be an effective approach to eradicate cancer cells by triggering ferroptosis [30]. It remains unknown whether the NRF2-SLC7A11-ferroptosis pathway coordinates with nucleolar stress to regulate cancer cell survival and growth.

In our attempt to address this question, we identified UTP11 as a novel regulator of nucleolar stress and NRF2-dependent ferroptosis. The UTP (U-three small nucleolar RNA-associated protein) family proteins were found to participate in the biosynthesis of 18S rRNA as components of the small subunit (SSU) processome in yeast [31]. Human UTP11 was found to be a potential prognostic biomarker in hepatocellular carcinoma [32,33]. However, the role of UTP11 in cancer development and the underlying mechanism remain largely elusive. In this study, we showed that UTP11 is highly expressed and associated with poor prognoses in multiple human cancers. Ablation of UTP11 triggers nucleolar stress, leading to p53 activation and cancer cell growth arrest *in vitro* and in vivo because it is essential for pre-rRNA processing by binding to the pre-rRNA processing factor MPP10, also known as MPHOSPH10. Surprisingly, UTP11 deficiency also elicits p53-independent inhibition of cancer cell survival. Interestingly, UTP11 can elongate NRF2 mRNA's half-life and enhance its expression by binding to its transcripts. By contrast, depletion of UTP11 represses SLC7A11 transcription by destabilizing NRF2 mRNA, thereby promoting ferroptosis of cancer cells independently of p53. Altogether, our findings unveil UTP11 as a new regulator of the nucleolar stress-p53 and SLC7A11-ferroptosis pathways and as an oncogenic protein critical for cancer cell survival. Our results also suggest that targeting UTP11 might be an attractive strategy to eliminate cancer cells by inducing nucleolar stress and ferroptosis.

## 2 Materials and methods

### 2.1 Cell culture and transient transfection

Human cancer cell lines, CAL-51, MCF-7, HCT116 ^p53+/+^, HCT116 ^p53−/−^, and RKO, were cultured in Dulbecco's modified Eagle's medium supplemented with 10% fetal bovine serum, penicillin (100 U/ml) and streptomycin (0.1 mg/ml), and incubated at 37 °C in a humidified incubator containing 5% CO2. Plasmids and siRNAs were transiently transfected into cells that were seeded on the plate overnight as indicated in figure legends using Hieff Trans Liposomal transfection reagent following the manufacturer's protocol (Yeasen, Shanghai, China). Cells were harvested at 36–72 h post transfection for future experiments. The cycloheximide (CHX) and proteasome inhibitor MG132 were purchased from MedChemExpress (Shanghai, China).

### 2.2 Plasmids and antibodies

The plasmids encoding HA-MDM2, p53, His-Ub were described previously [34]. The Flag-tagged pENTER-UTP11 plasmid was purchased from Vigene Biosciences (Shandong, China). Human UTP11 and MPP10 were subcloned into the vectors of Flag-pcDNA3.1 and Myc-His-pcDNA3.1, respectively. The primers for subcloning are listed in Supplementary Table 7. The anti-Flag (Cat. No. F1804, Sigma-Aldrich, St louis, MO, USA), anti-HA (Cat. No. 2367, Cell Signaling Technology, Danvers, MA, USA), anti-UTP11 (Cat. No: #46701, Signalway Antibody), anti-p53 (Cat. No. sc-126, DO-1, Santa Cruz Biotechnology), anti-MDM2 (Cat. No. ab16895, 2A10, Abcam), anti-GAPDH (Cat. No. 60004-1-Ig, Proteintech), anti-RPL5 (Cat. No. ab86863, Abcam), anti-RPL11 (Cat. No. ab79352, Abcam), anti-p21 (Cat. No. 2947, Cell Signaling Technology), anti-B23 (Cat. No. sc-271737, Santa Cruz BioteChnology), anti-MPP10 (Cat. No. ER65428, Hangzhou HuaAn Biotechnology), anti-SLC7A11 antibody (Cat. No.26864-1-AP, Proteintech), anti-NRF2 (Cat. No.16396-1-AP, Proteintech), and anti-vinculin antibody (Cat. No.13901, Cell Signaling Technology) were commercially purchased. The secondary antibodies used were HRP-conjugated affinipure goat anti-rabbit IgG (Cat. No. SA00001-2, Proteintech) and anti-mouse IgG (Cat. No. SA00001-1, Proteintech). Proteins were visualized with the ECL chemiluminescence reagent (Yeasen).

### 2.3 RNA-sequencing

CAL-51 cells transfected with siNC or siUTP11 for 48 h were collected, total RNA was isolated using RNAiso Plus following the manufacturer's protocol (Takara, Japan), and RNA-sequencing was provided by OEbiotech (shanghai, China).

### 2.4 Reverse transcription and quantitative real-time PCR

Total RNA was isolated using RNAiso Plus (Takara, Japan). Complementary DNAs (cDNAs) were synthesized from 0.2 to 0.5 μg RNA using Hiscript III qRT SuperMix (Vazyme). Quantitative PCR (qPCR) was conducted using SYBR qPCR Master Mix according to the manufacturer's protocol (Vazyme). The relative expression levels of mRNAs were calculated using the comparative Ct method normalized to GAPDH. The primers for Quantitative PCR (qPCR) are listed in Supplementary Table 6.

### 2.5 Northern blotting

Northern blotting assay was performed following the manufacturer's protocol of NorthernMax-Gly Kit (Thermo Fisher Scientific, USA). Briefly, RNA (30–60 μg) was denatured with an equal volume of Glyoxal Load Dye for 30 m at 50 °C and loaded on 1% agarose gel for electrophoresis in 1 × Gel Prep/Running buffer at 60 V for 2 h. After electrophoresis, RNA was transferred on BrightStar™-Plus nylon membrane (Thermo Fisher Scientific, USA) using transfer buffer for 3 h, and transferred RNA was ultraviolet-crosslinked (at 254 nm) at 1.5 J cm^2^. Then, pre-hybridization was performed at 65 °C for 30 m and hybridization was performed at 42 °C overnight. The membrane was washed with Low Stringency Wash Solution for 10 m at room temperature, then with the same solution for 2 m at 42 °C. Subsequently, the membrane was blocked with blocking buffer for 30 m and incubated with anti-DIG for 1 h at room temperature (Universal Biotech Co, shanghai). The membrane was then washed with washing buffer twice for 15 m and with detecting buffer for 5 m. Finally, the membrane was developed with the CDP-Star (Roche, USA). The probe used in this study was described previously [35] and the digoxigenin (DIG)-labeled probe was synthesized by GENEWIZ (Suzhou, China).

### 2.6 Immunoblotting

Proteins were extracted in ice-cold lysis buffer [50 mM Tris/HCl (pH 7.5), 0.5% Nonidet P-40 (NP-40), 1 mM EDTA, 150 mM NaCl, 1 mM dithiothreitol (DTT), 0.2 mM phenylmethylsulfonyl fluoride (PMSF), 10 μM pepstatin A, 1 μg/ml leupeptin and 10% protease inhibitor cocktail]. Equal amounts of clear cell lysate (20–80 μg) were used for immunoblotting (IB) analysis as described previously [36]. Original IB blots for all relevant figures are shown in “Supplementary Material—Original Blots”.

### 2.7 Immunoprecipitation

CAL-51 cells were transfected with control or UTP11 siRNA for 48 h and treated with MG132 for 4–6 h before being harvested. Immunoprecipitation (IP) was performed using antibodies indicated in the figure legends as described previously [37]. Briefly, 500–1000 μg of proteins were incubated with the indicated antibody at 4 °C for 5 h. Protein A or G beads (Santa Cruz Biotechnology) were then added and the mixture was left to incubate at 4 °C for additional 2 h. The beads were washed 6–8 times with lysis buffer. Protein interactions were detected by IB as described above.

### 2.8 Immunofluorescence staining

HCT116 ^p53+/+^ and HEK293T cells transfected with siRNA and plasmids as indicated in the figure legends were fixed with methanol in −20 °C for overnight. The fixed cells were washed by phosphate-buffered saline (PBS) and blocked with 8% bovine serum albumin (BSA) in PBS for 1 h, then the cells were incubated with primary antibodies (anti-UTP11, 1:50 dilution; anti-NPM1, 1:50 dilution; anti-flag, 1:100; anti-Myc, 1:100) in 2% BSA at 4 °C for overnight. After that, the cells were washed with PBS and incubated with the corresponding Fluorescent secondary antibodies (Yeasen) and DAPI (Sigma-Aldrich). Images were acquired with inverted fluorescence microscope (Leica, Wetzlar, Germany).

### 2.9 In vivo ubiquitination assay

HCT116 ^p53−/−^ cells stably expressing shNC or shUTP11 were transfected with plasmids encoding p53, HA-MDM2 or His-Ub as indicated in the figure legend and treated with MG132 for 4–6 h before being harvested. At 48 h after transfection, cells were harvested and split into two aliquots, one for IB and the other for the ubiquitination assay. In brief, cell pellets were lysed in buffer I [8 M urea, 0.1 M Na_2_HPO_4_/NaH_2_PO_4_ (pH 8.0), 10 mM Tris-HCl (pH 8.0), 10 mM β-mercaptoethanol, and 5 mM Imidazole] and incubated with Ni-NTA beads (Takara) that capture His-tagged proteins/complex at room temperature for 4 h. Beads were washed twice with buffer I, then twice with buffer II [8 M urea, 0.1 M Na_2_HPO_4_/NaH_2_PO_4_ (pH 6.3), 10 mM Tris-HCl (pH 6.3), 10 mM β-mercaptoethanol]. The captured proteins were eluted and analyzed by IB with the indicated antibodies.

### 2.10 RNA interference and generation of stable cell lines

The siRNAs against UTP11, RPL5, and RPL11 were synthesized and purified by GenePharma (Shanghai, China). The siRNA sequences were 5′-GAAGCTAAGAAAATCGAAA-3’ (siUTP11-1), 5′-GGATGGAGTACATATTATT-3’ (siUTP11-2), 5′-GGAGGAGAUGUAUAAGAAA-3’ (siRPL5) and 5′-GGAACUUCGCAUCCGCAAA-3’ (siRPL11). siRNAs were introduced into cells using Hieff Trans Liposomal transfection reagent following the manufacturer's protocol (Yeasen). Cells were harvested 48–72 h after transfection for IB or RT-qPCR. The shRNA sequence, 5′-GAAGCTAAGAAAATCGAAA-3′, for UTP11 was obtained from Sigma-Aldrich and subcloned into the pLKO.1 vector. The shRNA plasmid along with the packaging plasmids, psPAX2 and pMD2.G, was introduced into HEK293T cells. The lentivirus particles were collected 48 h after transfection and then used for infection of CAL-51, HCT116 ^p53+/+^, and HCT116 ^p53−/−^cells. Stable cells were selected with 1 μg/ml puromycin.

### 2.11 Cell viability assay

Cell viability was evaluated by Cell Counting Kit-8 (CCK-8) assay according to the manufacturer's instructions (Dojindo). Cells of 2–3.5 × 10^3^ were seeded per well in 96-well culture plates in triplicate at 6–12 h post transfection. CCK-8 was added to each well at a final concentration of 10% at different time points as indicated and the absorbance of samples was measured at 450 nm using a Microplate Reader.

### 2.12 Colony formation assay

Cells of 1 × 10^3^ were plated in a 6 cm plate 6–18 h after transfection and cultured for 14 days. The medium was changed every 3 days until colonies were visible. The colonies were then fixed with methanol and stained with 0.2% crystal violet solution at RT for 30 m. The counts of colonies were quantified by ImageJ.

### 2.13 Cell cycle analysis

Cells transfected with siRNAs were fixed with 70% ethanol overnight and treated with 250 μl buffer (50 μg/ml RNase A, and 0.1% Triton X-100 in PBS) at 37 °C for 30 m. After that, the cells were stained with 250 μl buffer [50 μg/ml Propidium Iodide (PI) (Vazyme), and 0.1% Triton X-100 (Sangon) in PBS] for 30 m in the dark. In the end, the cell cycle was analyzed by flow cytometry (CytoFLEX S, Beckman Coulter, Indianapolis, IN, USA).

### 2.14 Transwell cell migration assay

The transwell chambers were inserted in a 24-well plate. Cells of 5–10 × 10^4^ suspended in 200 μl serum-free medium were added to top chambers. The lower chambers were filled with 800 μl 20% FBS culture medium. After culture for 36–48 h at 37 °C, cells on the upper surface were scraped and washed away, and cells on the lower surface were fixed with methanol and stained with 0.2% crystal violet. Migratory cells were counted in at least three randomly selected fields under an optical microscope and quantified by ImageJ.

### 2.15 Mouse xenograft study

Four-week-old female BALB/c nude mice were purchased from and fed in Laboratory Animal Science of Fudan University Shanghai Cancer Center. CAL-51 cells stably expressing shNC or shUTP11 [6 × 10^6^ cells suspended in DMEM with 50% Matrigel (BD Biosciences)] were injected into right flanks of mice. To verify whether UTP11 deficiency-mediated tumor-inhibitory effects are dependent on p53, we performed an additional set of experiments using 5 × 10^6^ HCT116 ^p53+/+^ and HCT116 ^p53−/−^ cells stably expressing shNC or shUTP11. Tumor growth was monitored with electronic digital calipers in two dimensions. Tumor volume was calculated with the formula: tumor volume (mm^3^) = (length × width^2^) × 0.52. Finally, Mice were killed by euthanasia and tumors were harvested for analysis. The animal protocols were in compliance with ethical guidelines and approved by the Animal Welfare Committee of Fudan University Shanghai Cancer Center.

### 2.16 Glutathione assay

Glutathione (GSH) levels were measured using the GSH kit according to the manufacturer's instructions (Cat. No. A006-1, Nanjing Jiancheng Bioengineering Institute). Cells transfected with siRNAs for 48 h were digested with trypsin and rinsed with precooled PBS. After that, cells were ultrasonicated in PBS for 2 m and centrifuged at 3500 rpm for 10 m, and the supernatant was mixed with corresponding reagents in the kit. The absorbance at 420 nm was measured by a microplate reader.

### 2.17 Malondialdehyde assay

Intracellular Malondialdehyde (MDA) levels were detected following the manufacturer's protocol of the Malondialdehyde (MDA) assay kit (DOJINDO, Shanghai). Briefly, cells transfected with UTP11 siRNAs or UTP11-encoding plasmids were harvested in antioxidant PBS solution, and suspended in lysis buffer and working solution. The mixture was incubated at 95 °C for 15 m, then ice-cooling for 5 m. The supernatant was collected by centrifugation at 10,000 g for 10 m. Finally, the fluorescence intensity was measured by a microplate reader at ex 540 nm and em 590 nm.

### 2.18 RNA immunoprecipitation

Cells transfected with empty vector or Flag-UTP11 were harvested and suspended in RIP buffer (10 mM Tris, 150 mM NaCl, 1 mM Na_2_EDTA^**.**^2H_2_O, 3.5 mM SDS, 1 mM DTT, 1% NP-40, pH 7.4). The cell lysate was then immunoprecipitated with anti-Flag magnetic beads (Cat. No. B26101, bimake, Shanghai, China) overnight at 4 °C. The beads were washed six times with RIP buffer, followed by RNA purification and RT-qPCR analysis. The primers for RIP-qPCR are listed in Supplementary Table 8.

### 2.19 RNA stability assay

To determine if UTP11 knockdown affects the stability of NRF2 mRNA in HCT116 ^p53−/−^ cells, we treated cells with 5 μg/ml actinomycin D (Cat. No. HY-17559, MedChemExpress) at different time points as indicated. Cells were then harvested for RNA isolation and RT-qPCR analysis.

### 2.20 Chromatin immunoprecipitation

Cells were crosslinked with 37% formaldehyde for 10 m at room temperature and neutralized with glycine to a final concentration of 0.2 M for 5 m. Cells were harvested after being washed three times with cold PBS, suspended in cell lysis buffer (50 mM Tris-HCl pH 7.5, 140 mM NaCl, 1 mM EDTA, 10% glycerol, 0.5% NP-40, 0.25% TritonX-100, and proteinase inhibitor cocktail), and incubated in ice for 30 min. Nuclei were resuspended in 0.5 ml nuclear lysis buffer (50 mM Tris-HCl pH 8.0, 10 mM EDTA, 1% SDS, and proteinase inhibitor cocktail). After sonication (60 cycles with 30 s on and 30 s off), lysates were centrifuged at 12000 g for 5 m, and the supernatants were mixed with anti-NRF2 or IgG for overnight and then with protein A/G beads for 2 h at 4 °C. Beads were washed sequentially with Low Salt Wash Buffer (50 mM Tris-HCl pH 8.0, 0.1% SDS, 0.5% deoxycholate, 1 mM EDTA, 1% NP-40, and 150 mM NaCl), High Salt Wash Buffer (50 mM Tris-HCl pH 8.0, 0.1% SDS, 0.5% deoxycholate, 1 mM EDTA, 1% NP-40, and 500 mM NaCl), LiCl Wash Buffer (50 mM Tris-HCl pH 8.0, 250 mM LiCl, 0.1% SDS, 0.5% deoxycholate, 1 mM EDTA, and 1%NP-40), and TE Buffer (10 mM Tris-HCl pH 8.0, and 1 mM EDTA). The protein-DNA complex was eluted with ChIP elution buffer (1% SDS and 0.1 M NaHCO_3_). After decrosslinking for 2 h at 62 °C, DNA was extracted and analyzed by qPCR. ChIP-PCR primers for SLC7A11 are 5′-TTACTACTTCTGGATTGGCTA-3′ and 5′-CTTGTATTTAAGCGCCTGCC-3’.

### 2.21 Human breast cancer and colorectal cancer specimens

A total of six pairs and ten pairs of breast cancer and adjacent normal tissues from the First Affiliated Hospital of China Medical University were used for IB and RT-qPCR analysis, respectively. Nineth-one paraffin-embedded sections of breast cancer obtained from the First Affiliated Hospital of China Medical University were subjected to IHC analysis. This study was approved by the Human Research Ethics Committee of the First Affiliated Hospital of China Medical University. In addition, a total of ten pairs of colorectal cancer and adjacent normal tissues and 150 paraffin-embedded sections of colorectal cancer obtained from the First Affiliated Hospital of Nanchang University were used for IHC analysis. This study was approved by the Human Research Ethics Committee of the First Affiliated Hospital of Nanchang University.

### 2.22 Immunohistochemistry

Paraffin-embedded sections of breast or colorectal cancer tissues were deparaffinized at 65 °C for 1–2 h and then subjected to xylene and a graded series of alcohol. Following that, the slides were heated with Sodium citrate-EDTA antigen repair solution (cat. No. P0086, Beyotime) for antigen unmasking. After cooling, the slides were incubated for 1–2 h with primary antibodies at room temperature. The sections were then covered with horseradish peroxidase (HRP)-conjugated secondary antibody (cat. No. GK500705, GeneTech) at RT for 30–60 m and incubated with 3′-diaminobenzidine (cat. No. GK500705, GeneTech) for 5 m. Subsequently, the slides were counterstained with hematoxylin, dehydrated with a graded series of alcohols, and mounted with coverslips and mounting medium. The staining density was measured using a Leica CCD camera DFC420 connected to a Leica DM IRE2 microscope (Leica Microsystems Imaging Solutions Ltd.). The IHC scores were measured by multiplying staining intensity (0 = no, 1 = weak, 2 = moderate, 3 = strong) with percentage of positive staining (0 = negative, 1 ≤ 10%, 2 = 10–50%, 3 ≥ 50%). UTP11 was defined as low expression when IHC scores were ≤4, and as high expression when IHC scores were >4.

### 2.23 Bioinformatic analysis

To explore the underlying biological mechanisms related to UTP family genes and p53 signaling, we extracted known protein-protein interacting relations between 27 UTP genes and 587 p53 signaling genes. In brief, we used Pathway Commons database (Version 12) [38] to create a non-redundant human interactome based on all known protein-protein interactions, which included 30918 genes and 1787402 gene-gene connections. Next, we used GenRev [39], a subnetwork extraction tool, to investigate the connections between UTP-related rRNA processing and 587 p53 signaling genes. A total of 627 genes related to UTP and p53 signaling were mapped to the PathCommons interactome first by using GenRev. The algorithm then connected the mapped genes to form a fully connected sub-network with as many input genes as possible.

### 2.24 Statistics

All *in vitro* experiments were performed in biological triplicate. P-values were obtained by *t*-test or analysis of variance using GraphPad Prism 5.0. The Kaplan-Meier method was used to analyze the significant difference of patient survival. p < 0.05 was considered statistically significant. Multivariate Cox proportional hazard models were used to calculate hazard ratios with 95% confidence intervals. Asterisks denote statistical significance: *p < 0.05; **p < 0.01; ***P < 0.001. Quantitative data are presented as mean ± SD.

## 3 Results

### 3.1 Identification of UTP11 as a potential oncoprotein in cancer

**Fig. 1:**
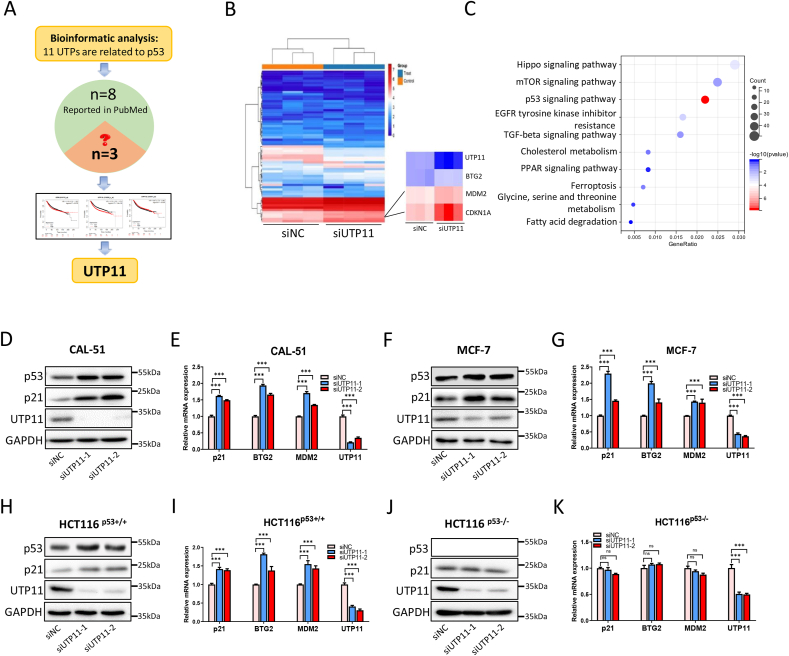
**Ablation of UTP11 induces p53 activation. (A)** Schematic diagram depicts the screening for UTP11. **(B)** The heatmap of RNA-sequencing analysis of CAL-51 breast cancer cells reveals that p53 target genes are induced by UTP11 knockdown. **(C)** Signaling pathways that are enriched in UTP11-depleted cells are displayed by Kyoto Encyclopedia of Genes and Genomes (KEGG) analysis. **(D**–**I)** Knockdown of UTP11 increases the expression of p53 and its target genes. CAL-51 (D, E), MCF-7 (F, G) and HCT116 ^p53+/+^ cells (H, I) were transfected with control or UTP11 siRNAs, followed by IB and RT-qPCR analyses. **(J, K)** Knockdown of UTP11 has no effect on the expression of p53 and its target genes in HCT116^p53−/^^−^ cells. ***p < 0.001.

Because of the essential role of UTP family members in ribosome biogenesis in yeast as mentioned above [31], we systematically investigated if their human orthologs might regulate p53 activity or cell growth. By dissecting protein-protein interaction networks [38,39] (Supplementary Fig. 1A; Supplementary Table 1) and reviewing literatures (Supplementary Fig. 1B), we found that 11 out of 27 UTP proteins are connected to the p53 signaling pathway or cell growth. However, three of the 11 UTP proteins, including UTP6, UTP11, and UTP14C, have not yet been studied for their ability to regulate p53 activity and cancer development (Fig. 1A). Our analysis of the prognostic significance of the three UTPs in breast cancer revealed that only UTP11 is highly associated with the poor prognosis with a *p*-value of 1.8E-09 (Supplementary Fig. 1C). Through our analysis of TCGA database, we also found that the *UTP11* gene is amplified in human cancers (Supplementary Fig. 2A). In addition, both mRNA and protein levels of UTP11 were upregulated in different types of cancerous tissues compared with normal tissues (Supplementary Figs. 2B–2F). Moreover, higher levels of UTP11 were associated with worse prognoses in various cancers (Supplementary Figs. 2G–2K). These results suggest that UTP11 may act as an oncogenic protein in cancer. Thus, we decided to further investigate this protein.

### 3.2 Ablation of UTP11 induces p53 activation

First, we determined whether *UTP11* may play an oncogenic role by suppressing p53 activity. To test this idea, we performed an RNA-sequencing (RNA-seq) analysis of CAL-51 breast cancer cells. As a result, knockdown of UTP11 led to the significant increase of multiple p53 target genes at their RNA levels (Fig. 1B). This was consistent with the Kyoto Encyclopedia of Genes and Genomes (KEGG) analysis (Fig. 1C). To confirm this result, we performed RT-qPCR and immunoblotting (IB) analyses and found that ablation of UTP11 by two independent siRNAs markedly increases the expression of p53 and p21 at protein levels as well as several target genes at RNA levels, such as *p21*, *BTG2*, and *MDM2*, in both CAL-51 and MCF-7 breast cancer cell lines (Fig. 1D–G). This increase was p53-dependent, as UTP11 knockdown only significantly induced the expression of p53 target genes in HCT116 ^p53+/+^ (Fig. 1H and I), but not in HCT116 ^p53−/−^ cells (Fig. 1J and K). Notably, UTP11 knockdown barely affected levels of p53 mRNA as determined by RT-qPCR (Supplementary Figs. 3A–3C), but markedly upregulated protein levels of p53 (Fig. 1D, F, and 1H). Conversely, ectopic expression of UTP11 reduced p53 protein levels (Supplementary Figs. 3D and 3F) and the expression of its target genes (Supplementary Figs. 3E and 3G). These results indicate that UTP11 deficiency leads to the activation of the p53 pathway and suggest that UTP11's potential oncogenic role might be executed in part via suppression of p53.

### 3.3 UTP11 is required for 18S rRNA synthesis by binding to MPP10

**Fig. 2:**
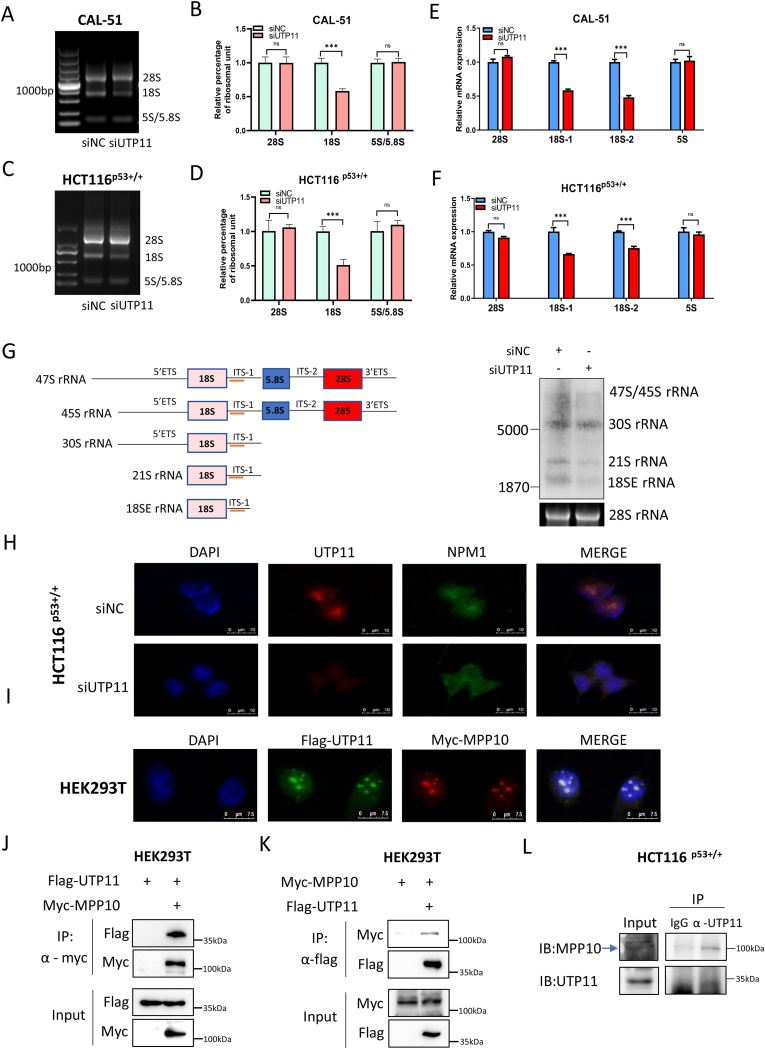
**UTP11 is required for 18S rRNA synthesis by binding to MPP10. (A**–**D)** Knockdown of UTP11 reduces the production of 18S rRNA. CAL-51 and HCT116 ^p53+/+^ cells were transfected with control or UTP11 siRNA, followed by agarose gel electrophoresis (A, C) and quantification by densitometry (B, D). **(E**–**F)** Knockdown of UTP11 selectively reduces levels of 18S rRNA. Fragments of 18S or 28S rRNA were detected by RT-qPCR in CAL-51 (E) and HCT116 ^p53+/+^ cells (F) transfected with control or UTP11 siRNA. **(G)** Knockdown of UTP11 reduces the formation of 18S rRNA processing intermediates 21S and 18SE RNA. Cells were transfected with control or UTP11 siRNA, followed by Northern blotting analysis. A schematic illustration of 18S rRNA processing with the probe (orange) used is shown in the left panel. **(H)** UTP11 depletion disrupts the nucleolar localization of NPM1. Cells were transfected with control or UTP11 siRNA, followed by IF staining. **(I)** UTP11 and MPP10 are co-localized in the nucleolus. Cells were transfected with Flag-UTP11 and Myc-MPP10, followed by IF staining using anti-Flag and anti-Myc antibodies. **(J, K)** UTP11 interacts with MPP10. Cells were transfected with the indicated plasmids, followed by co-IP-IB analysis using antibodies as indicate. **(L)** Endogenous interaction of UTP11 and MPP10. HCT116 ^p53+/+^ cells were treated with MG132 for 6 h and subjected to co-IP-IB analysis. ***p < 0.001. (For interpretation of the references to color in this figure legend, the reader is referred to the Web version of this article.)

Though Utp11 has been shown to play a role in ribosome biogenesis in yeast [31], it remained untested if this role is conserved in the regulation of ribosome biogenesis in human cells. To test this, we first examined if UTP11 is required for ribosome biogenesis in breast and colorectal cancer cells. The gel electrophoresis analysis of rRNA products showed that UTP11 knockdown significantly reduces 18S rRNA production without affecting levels of 28S and 5.8S/5S rRNAs in CAL-51 (Fig. 2A and B) and HCT116 ^p53+/+^ cells (Fig. 2C and D). This result was confirmed by RT-qPCR analysis with specific primers for 18S or 28S rRNAs by RT-qPCR, as knockdown of UTP11 selectively reduced levels of 18S, but not 28S, rRNA (Fig. 2E and F). In addition, we performed Northern blotting analysis and found that UTP11 knockdown dramatically impairs the formation of 18S rRNA processing intermediates 21S and 18SE RNA (Fig. 2G), which is consistent with the function of the SSU processome in yeast [31,40]. Since impairment of rRNA synthesis could cause disruption of nucleolar architecture [7,8], we next tested if UTP11 knockdown can do the same by performing immunofluorescence (IF) staining of a nucleolar marker, NPM1. As expected, NPM1 was mainly detected in the nucleolus under the normal culture condition, but ablation of UTP11 led to its relocation to the nucleoplasm of HCT116 cells (Fig. 2H), indicating that UTP11 deficiency might induce nucleolar stress. The U3-specific protein Mpp10, which is a critical component of the SSU processome, has been shown to associate with Utp proteins to facilitate 18S rRNA biogenesis in yeast [31]. We wondered if UTP11 interacts with MPP10 in human cells. To this end, we performed IF staining of UTP11 and MPP10 by exogenously overexpressing both the proteins in 293 cells. The results showed that UTP11 and MPP10 are co-localized in the nucleolus (Fig. 2I). Their association in human cancer cells was further validated by a set of co-immunoprecipitation (co-IP) assays. Flag-tagged UTP11 and Myc-tagged MPP10 bound to each other, as measured by reciprocal co-IP assays (Fig. 2J and K). Consistently, the endogenous complex of these two proteins was also detected by co-IP analysis (Fig. 2L). Together, these results demonstrate that UTP11 interacts with MPP10 in the nucleoli and is crucial for 18S rRNA biosynthesis in human cells.

### 3.4 Ablation of UTP11 stabilizes p53 through RPL5/RPL11 inhibition of MDM2

**Fig. 3:**
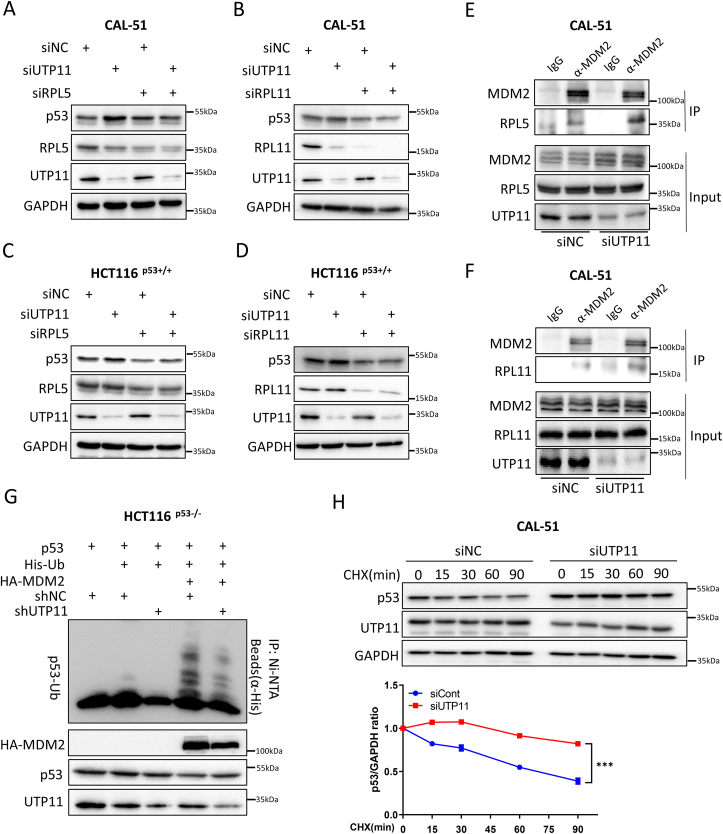
**Ablation of UTP11 stabilizes p53 through RPL5/RPL11 inhibition of MDM2. (A**–**D)** Knockdown of RPL5 or RPL11 compromises the induction of p53 by UTP11 depletion. CAL-51 (A, B) and HCT116 ^p53+/+^ cells (C, D) were transfected with control, UTP11 siRNA, RPL5 siRNA, and RPL11 siRNA as indicated. Cell lysates were subjected to IB analysis with indicated antibodies. **(E, F)** RPL5-MDM2 and RPL11-MDM2 interactions are increased by depletion of UTP11. CAL-51 cells were transfected with control or UTP11 siRNA, followed by co-IP-IB assays using antibodies as indicated. The proteasome inhibitor MG132 was supplemented into medium for 5 h before cell harvest. **(G)** Knockdown of UTP11 diminishes MDM2-induced p53 ubiquitination. HCT116 ^p53−/^^−^ cells stably expressing control or UTP11 shRNA were transfected with plasmids encoding p53, His-Ub, and HA-MDM2 as indicated and treated with MG132 for 5 h, followed by in vivo ubiquitination assay and IB analysis. **(H)** UPT11 knockdown extends the half-life of p53 protein. CAL-51 cells were transfected with control or UTP11 siRNA. CHX (100 mg/ml) was supplemented into medium for the indicated time before cells were harvested for IB analysis. ***p < 0.001.

It has been well-documented that numerous RPs are released from the nucleolus into the nucleoplasm to interact with MDM2 and inhibit MDM2-mediated p53 degradation upon nucleolar stress [7,8]. Among these RPs, RPL5 and RPL11 are the most important in the regulation of the MDM2-p53 circuit, as the impairment of RPL5/RPL11 interactions with MDM2 completely abrogated p53 activation in response to nucleolar stress [[15], [16], [17],^21^]. Thus, we tested whether RPL5 and RPL11 are required for UTP11 depletion-induced p53 activation. Knockdown of UTP11 upregulated p53 levels, whereas this upregulation was dramatically impaired upon further depletion of RPL5 or RPL11 in CAL-51 cells (Fig. 3A and B). Consistently, ablation of RPL5 or RPL11 also impaired p53 activation that is caused by depleting UTP11 in HCT116 ^p53+/+^ cells (Fig. 3C and D). These results indicate that the two RPs are critical for UTP11 depletion-induced p53 activation. We noticed that UTP11 knockdown also moderately reduces both RPL5 and RPL11 levels in CAL-51 cells (Fig. 3A and B). This is possibly because ribosomal components may associate with and stabilize each other as previously reported [17,41]. In addition, we tested whether UTP11 knockdown increases interactions between RPL5/RPL11 and MDM2. As anticipated, ablation of UTP11 dramatically enhanced MDM2 binding to the two RPs, as evidenced by an increase in the amounts of RPL5 and RPL11 that were co-immunoprecipitated with MDM2 (Fig. 3E and F). Since ectopic expression of RPL5 and RPL11 represses MDM2-mediated p53 ubiquitination [[9], [10], [11]], we tested if UTP11 knockdown can do the same. As shown in Fig. 3G, the overexpression of MDM2 increased p53 ubiquitination, whereas this ubiquitination was markedly reduced when UTP11 was knocked down. Consistently, UPT11 knockdown prolonged the half-life of p53 protein, as measured by a cycloheximide-chase assay (Fig. 3H). These results demonstrate that UPT11 deficiency causes nucleolar stress, consequently releasing RPL5 and RPL11 that stabilize p53 by inhibiting MDM2 activity.

### 3.5 UTP11 deficiency inhibits breast cancer cell growth and migration

**Fig. 4:**
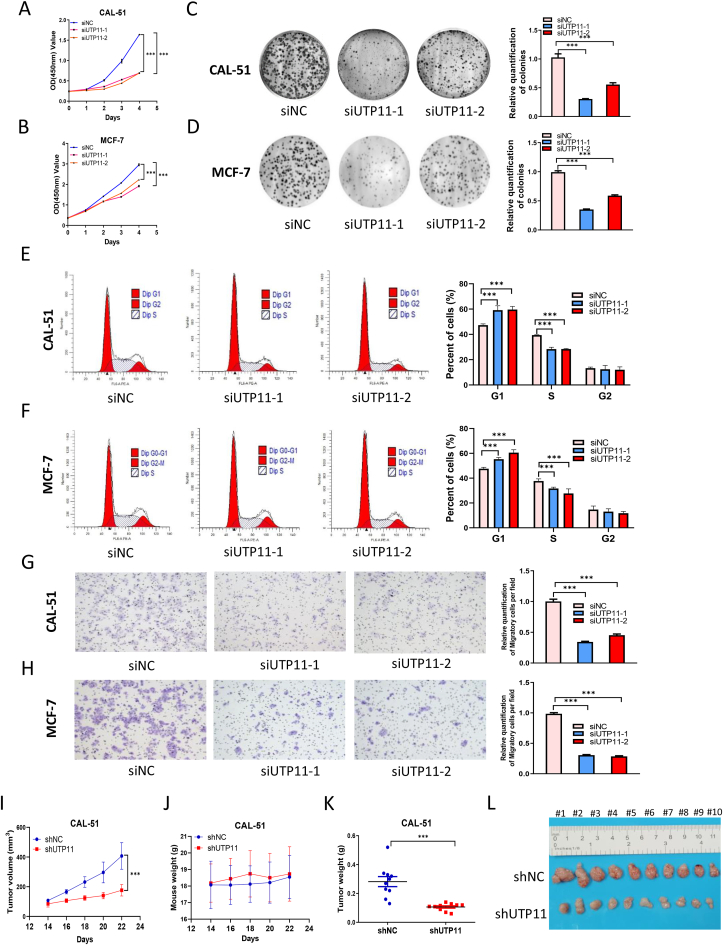
**UTP11 deficiency inhibits breast cancer cell growth and migration. (A, B)** Knockdown of UTP11 suppresses proliferation of wild-type p53-harboring cancer cells. CAL-51 (A) and MCF-7 cells (B) were transfected with control or UTP11 siRNAs for 6–12 h and seeded in 96-well plates for cell viability assay. (**C, D)** Knockdown of UTP11 inhibits clonogenic ability of wild-type p53-harboring cancer cells. CAL-51 (C) and MCF-7 cells (D) were seeded in 6-well plates for about 14 days. Colonies were fixed with methanol, and visualized by crystal violet staining. **(E, F)** Knockdown of UTP11 induces G1 cell cycle arrest in wild-type p53-harboring cancer cells. CAL-51 (E) and MCF-7 cells (F) were transfected with control or UTP11 siRNAs, followed by flow cytometry analysis. **(G, H)** Knockdown of UTP11 impede migration of wild-type p53-harboring cancer cells. CAL-51 (G) and MCF-7 cells (H) were transfected with control or UTP11 siRNAs, followed by transwell cell migration assay. **(I**–**L)** UTP11 deficiency suppresses cancer growth in vivo. Depletion of UTP11 suppresses CAL-51 cell-derived xenograft tumor growth rate (I), weight (K), and size (L), while has no effect on mouse body weight (J). The data are represented as mean ± SD, n = 10. ***p < 0.001. (For interpretation of the references to color in this figure legend, the reader is referred to the Web version of this article.)

Since UTP11 is highly expressed in cancer (Supplementary Figs. 2A–2F) and its depletion triggers nucleolar stress and p53 activation (Fig. 1, Fig. 2, Fig. 3), we next determined whether depleting UTP11 might suppress the growth and migration of wild-type p53-harboring cancer cells. Knockdown of UTP11 using two independent siRNAs significantly inhibited the viability (Fig. 4A and B) and colony-formation (Fig. 4C and D) of CAL-51 and MCF-7 breast cancer cells. Also, ablation of UTP11 induced G1 cell cycle arrest of both breast cancer cell lines (Fig. 4E and F), which was likely due to the upregulation of p21 (Fig. 1D–I). Moreover, UTP11 ablation dramatically suppressed breast cancer cell migration, as determined by transwell assays (Fig. 4G and H). Consistent with these cell-based results, depleting UTP11 suppressed breast cancer growth in vivo. CAL-51 cells stably expressing control or UTP11 shRNA were subcutaneously injected into flanks of nude mice. UTP11 deficiency dramatically reduced the growth rate of xenograft tumors (Fig. 4I) without significantly affecting mouse weight (Fig. 4J). The tumor weight and size were also decreased in response to UTP11 depletion (Fig. 4K and L). On the contrary, UTP11 overexpression significantly promoted the growth (Supplementary Figs. 4A–4D), colony formation (Supplementary Figs. 4E and 4F), and migration (Supplementary Figs. 4G and 4H) of cancer cells. These results demonstrate that depleting UTP11 suppresses cancer growth *in vitro* and in vivo, and suggest that UTP11 may be a potential therapeutic target for cancer treatment.

### 3.6 UTP11 deficiency-mediated tumor-inhibitory effects are partially dependent on p53

**Fig. 5:**
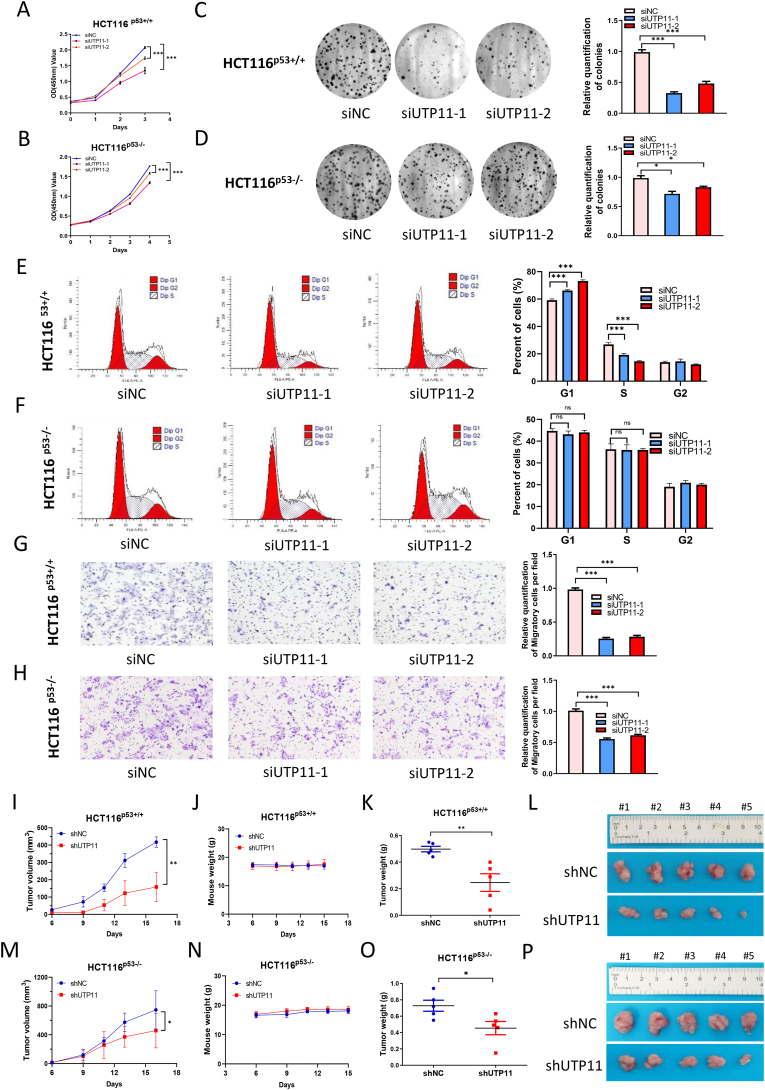
**UTP11 deficiency-mediated tumor-inhibitory effects are partially dependent on p53. (A, B)** Knockdown of UTP11 restrains proliferation of HCT116^p53+/+^ cells (A) more dramatically than HCT116 ^p53−/^^−^ cells (B). Cells were transfected with control or UTP11 siRNAs for 6–12 h and seeded in 96-well plates, followed by cell viability assay. **(C, D)** UTP11 depletion reduces the colony-forming ability of HCT116 ^p53+/+^ cells (C) more dramatically than HCT116 ^p53−/^^−^ cells (D). Cells were seeded in 6-well plates for about 14 days. Colonies were fixed with methanol, and visualized by crystal violet staining. (**E, F)** UTP11 deficiency induces G1 arrest in HCT116 ^p53+/+^ (E) but not HCT116 ^p53−/−^cells (F). Cells were transfected with control or UTP11 siRNAs, followed by flow cytometry analysis. **(G, H)** Knockdown of UTP11 impedes migration of HCT116 ^p53+/+^ cells (G) more dramatically than HCT116 ^p53−/^^−^ cells (H). **(I–P)** UTP11 depletion suppresses tumor growth partially dependently on p53 in vivo. UTP11 depletion dramatically suppresses HCT116 ^p53+/+^ cell-derived tumor growth rate (I), weight (K), and size (L), while also moderately reduces HCT116 ^p53−/^^−^ cell-derived tumor growth rate (M), weight (O), and size (P). Mouse body weight was not affected (J, N). The data are represented as mean ± SD, n = 5. *p < 0.05, **p < 0.01, ***p < 0.001. (For interpretation of the references to color in this figure legend, the reader is referred to the Web version of this article.)

Next, we examined whether the tumor-regressive outcomes of depleting UTP11 as shown above were p53-dependent. To test this idea, we employed the isogenic colorectal cancer cell lines, HCT116 ^p53+/+^ and HCT116 ^p53−/−^. Consistent with the results in breast cancer, UTP11 knockdown significantly inhibited HCT116 ^p53+/+^ cell growth and migration, as determined by cell viability (Fig. 5A), colony formation (Fig. 5C), and transwell assays (Fig. 5G). Intriguingly, these effects were partially dependent on p53, as UTP11 knockdown also reduced HCT116 ^p53−/−^ cell growth and migration, though to a lesser extent than those in HCT116 ^p53+/+^ cells (Fig. 5B, D, and 5H). These results suggest that a p53-independent mechanism may contribute to the effect of depleting UTP11 in the cells. However, the cell cycle arrest caused by depleting UTP11 appeared to be p53-dependent, as UTP11 depletion induced G1-arrest only in HCT116 ^p53+/+^ (Fig. 5E) cells, but not in HCT116 ^p53−/−^ cells (Fig. 5F). This must be due to p53-dependent upregulation of p21 and other cell cycle regulatory genes (Fig. 1D–K). Also, we found that UTP11 deficiency suppresses the growth of HCT116 ^p53+/+^ cell-derived xenograft tumors more markedly (Fig. 5I–L) than that of HCT116 ^p53−/−^ cell-derived tumors (Fig. 5M − P). Taken together, these results indicate that depleting UTP11 leads to the suppression of cancer development through p53-dependent and independent mechanisms.

### 3.7 UTP11 deficiency triggers ferroptosis via the NRF2-SLC7A11 axis

**Fig. 6:**
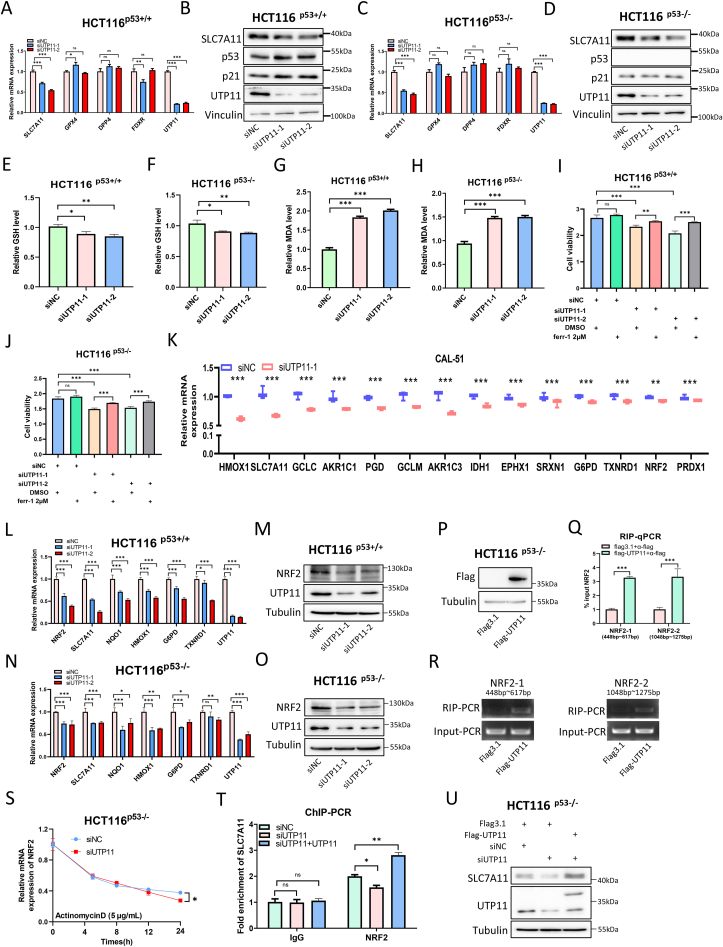
**UTP11 deficiency triggers ferroptosis by regulating the NRF2-SLC7A11 axis.** (**A-D)** Knockdown of UTP11 represses SLC7A11 mRNA and protein expression independently of p53. HCT116 ^p53+/+^ (A, B) and HCT116 ^p53−/^^−^ cells (C, D) transfected with the indicated siRNAs were subjected to RT-qPCR and IB analyses. **(E, F)** UTP11 depletion reduces GSH levels. HCT116 ^p53+/+^ (E) and HCT116 ^p53−/-^ cells (F) were transfected with the indicated siRNAs, followed by GSH assay. **(G, H)** UTP11 depletion elevates MDA levels. HCT116 ^p53+/+^ (G) and HCT116 ^p53−/-^ cells (H) were transfected with the indicated siRNAs, followed by MDA assay. **(I, J)** UTP11 depletion-caused cell growth inhibition is partially restored by Ferrostatin-1. HCT116 ^p53+/+^ (I) and HCT116^p53−/^^−^ cells (J) transfected with the indicated siRNAs were seeded in 96-well plates and treated with DMSO or Ferrostatin-1 (2 μM) for 48 h, followed by cell viability assay. **(K)** RNA-sequencing results reveal that knockdown of UTP11 reduces the expression of NRF2 and its target genes. **(L**–**O)** UTP11 depletion markedly represses NRF2 and its target gene expression. HCT116 ^p53+/+^ (L, M) and HCT116 ^p53−/-^ cells (N, O) transfected with the indicated siRNAs were subjected to RT-qPCR and IB analyses. **(P**–**R)** UTP11 interacts with NRF2 mRNA. HCT116 ^p53−/-^ cells were transfected with control or Flag-UTP11, and input samples were confirmed by IB (P). RNA immunoprecipitation (RIP) assays were performed to detect the interactions between UTP11 and NRF2 mRNA, followed by RT-qPCR (Q) and agarose gel electrophoresis (R). Two pairs of primers were designed to amplify fragments from 448 to 617 bp and 1048–1275 bp of NRF2 mRNA. **(S)** UTP11 depletion decreases NRF2 mRNA stability. HCT116 ^p53−/-^ cells transfected with control or UTP11 siRNA were treated with actinomycin D (5 μg/ml) for the indicated time, followed by RT-qPCR analysis. **(T, U)** UTP11 deficiency prevents NRF2 recruitment on SLC7A11 promoter. HCT116 ^p53−/-^ cells were transfected with siRNAs or plasmids as indicated, followed by ChIP- qPCR (T) and IB (U) analyses. *p < 0.05, **p < 0.01, ***p < 0.001.

The finding that UTP11 deficiency could lead to inhibition of the growth of p53-null cancer cells prompted us to explore a possible p53-independent mechanism. We re-analyzed RNA-seq data and found that UTP11 depletion is associated with alterations of some ferroptosis-associated genes (Fig. 1C; Supplementary Fig. 5A). Among them, *SLC7A11* was selected for further investigation as a top candidate gene (Supplementary Fig. 5A). To validate if depleting UTP11 could lead to the downregulation of SLC7A11, we performed IB and RT-qPCR assays and found that UTP11 knockdown significantly and consistently reduces mRNA and protein levels of SLC7A11 in HCT116 ^p53+/+^ (Fig. 6A and B), CAL-51 (Supplementary Figs. 5B and 5C), MCF-7 (Supplementary Figs. 5D and 5E), and RKO (Supplementary Figs. 5F and 5G) cells, as well as in HCT116 ^p53−/−^ cells (Fig. 6C and D). Conversely, ectopic expression of UTP11 increased both mRNA and protein levels of SLC7A11 in HCT116 ^p53+/+^ (Supplementary Figs. 5H and 5I) and HCT116 ^p53−/−^ cells (Supplementary Figs. 5J and 5K). These results suggest that the regulation of SLC7A11 expression by UTP11 is p53-independent. Considering SLC7A11 is critical for the uptake of cystine and synthesis of GSH [26], we tested if UTP11 might affect GSH levels in cancer cells. Indeed, ablation of UTP11 significantly reduced GSH levels (Fig. 6E and F; Supplementary Fig. 5L), whereas ectopic UTP11 increased GSH levels in both p53-positive and p53-null cancer cells (Supplementary Figs. 5M and 5N). The reduction in GSH levels can lead to an increase in reactive oxygen species (ROS) and induce ferroptosis [30]. Thus, we determined the intracellular levels of MDA, a product of lipid peroxidation that is proportional to the extent of ferroptosis [42]. As expected, MDA levels were elevated in response to UTP11 depletion (Fig. 6G and H; Supplementary Fig. 5O), but were reduced by UTP11 overexpression (Supplementary Figs. 5P and 5Q). In addition, we showed that knockdown of UTP11 inhibits the growth of HCT116 ^p53+/+^, HCT116 ^p53−/−^, and CAL-51 cells (Fig. 6I and J; Supplementary Fig. 5R), which is consistent with the results as shown in Fig. 4, Fig. 5B. Importantly, treatment of the cells with a specific ferroptosis inhibitor, Ferrostatin-1, partially rescued this inhibition (Fig. 6I and J; Supplementary Fig. 5R). These results indicate that UTP11 deficiency can trigger ferroptosis by downregulating SLC7A11 expression regardless of p53 status.

We then sought to dissect how UTP11 might regulate SLC7A11 expression. NRF2 is the master transcription factor that regulates the expression of a myriad of antioxidant genes in response to oxidative stress [43]. It has been reported that inhibition of NRF2 elicits p53-independent ferroptosis by diminishing SLC7A11 expression [29,44]. Thus, we tested if UTP11 might regulate SLC7A11 expression through NRF2. Our initial RNA-seq data suggested that UTP11 depletion could lead to reduced expression of NRF2 and its target genes (Fig. 6K). To validate this result, we performed IB and RT-qPCR analyses of protein and RNA levels of NRF2 and its target genes. Ablation of UTP11 markedly repressed NRF2 expression at both mRNA and protein levels in multiple cancer cell lines (Fig. 6L–O; Supplementary Fig. 5S-5U). Correspondingly, several NRF2 target genes, including SLC7A11, were also downregulated in UTP11-depleted cancer cells regardless of p53 status (Fig. 6L and N; Supplementary Figs. 5S and 5U). In addition, ectopic UTP11 increased both mRNA and protein levels of NRF2 independently of p53 (Supplementary Figs. 5H–5K). Since UTP11 possesses RNA-binding ability [31], we tested if UTP11 could bind to NRF2 mRNA and regulate its stability by conducting a set of RNA immunoprecipitation (RIP) and RT-qPCR assays with two pairs of primers. Indeed, ectopic UTP11 bound to NRF2 mRNAs (Fig. 6P–R). Also, UTP11 knockdown significantly reduced NRF2 mRNA levels when DNA transcription was blocked by 5 μg/ml actinomycin D, indicating that UTP11 is required for NRF2 mRNA stability (Fig. 6S). Furthermore, we tested whether UTP11 influences the recruitment of NRF2 to the *SLC7A11* promoter by conducting a chromatin immunoprecipitation (ChIP) assay. We showed that UTP11 depletion reduces the association of NRF2 with the *SLC7A11* promoter, whereas the overexpression of UTP11 increases NRF2 binding to SLC7A11 promoter (Fig. 6T). This result was further verified by IB analysis of the input samples, as UTP11 knockdown repressed, while its overexpression elevated, the expression of SLC7A11 (Fig. 6U). Collectively, these results demonstrate that UTP11 deficiency inhibits SLC7A11 expression by reducing the half-life of NRF2 mRNA.

### 3.8 UTP11 overexpression is correlated with poor prognoses in breast and colorectal cancers

**Fig. 7:**
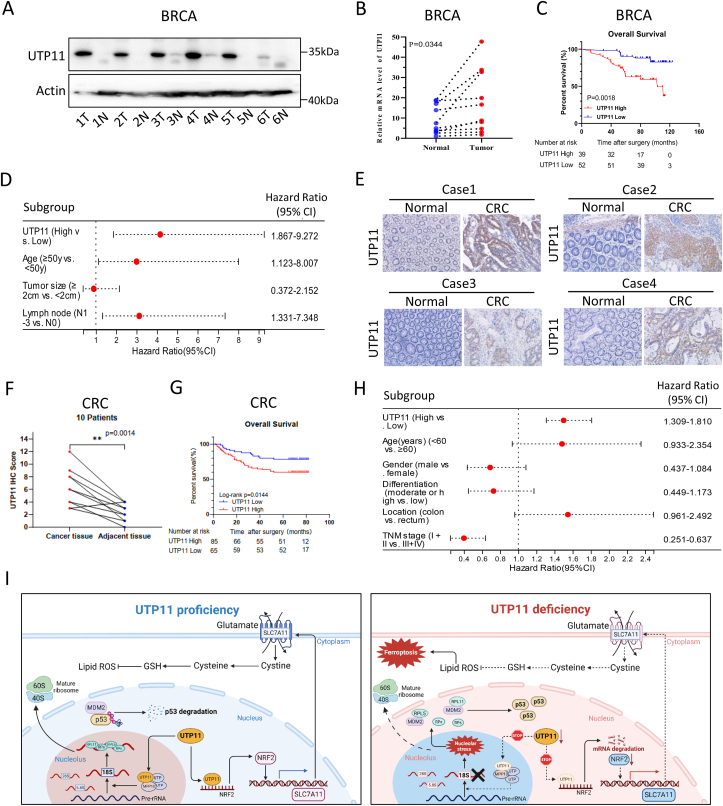
**UTP11 overexpression is correlated with unfavorable prognoses in breast and colorectal cancers (A)** UTP11 protein expression is upregulated in breast cancer samples compared to adjacent normal tissues. Six pairs of tissues were analyzed by IB. **(B)** UTP11 mRNA expression is upregulated in breast cancer samples compared to adjacent normal tissues. Ten pairs of tissues were analyzed by RT-qPCR. **(C, D)** Higher expression of UTP11 is significantly associated with shorter overall survival in a cohort of 91 breast cancer patients. **(E, F)** UTP11 protein expression is higher in colorectal cancer samples than adjacent normal tissues. Representative images (E) and the graph of ten pairs of samples (F) are shown. **(G, H)** Higher levels of UTP11 are associated with worse prognoses in 150 colorectal cancer patients. **(I)** A schematic diagram for UTP11 function in cancer. In UTP11-proficient cells, p53 is degraded through MDM2-mediated ubiquitination and lipid ROS is restricted via NRF2 antioxidant signaling pathway (e.g., SLC7A11), which promotes cancer cell survival and growth (left panel). In UTP11-deficient cells, the biosynthesis of 18S rRNA is impaired, resulting in nucleolar stress-induced p53 stabilization and activation. Additionally, UTP11 depletion promotes the destabilization of NRF2 mRNA, leading to the downregulation of SLC7A11 and increased ferroptosis (right panel). **p < 0.01.

Since UTP11 is essential for the growth and survival of breast and colorectal cancer cells as described above, we investigated the clinical significance of UTP11 overexpression in these two types of cancer. First, UTP11 expression was determined in breast cancer and paired normal tissues by IB and RT-qPCR analyses. Both the protein and mRNA levels of UTP11 were upregulated in breast cancer tissues compared with normal tissues (Fig. 7A and B). It should be noted that some normal tissues might contain a mixture of epithelial and adipose cells. In addition, immunohistochemistry (IHC) staining of UTP11 in 91 breast cancer specimens revealed that higher levels of UTP11 are significantly associated with higher tumor/node/metastasis (TNM) stages and worse overall survival of patients (Fig. 7C and Supplementary Table 2). Moreover, univariate and multivariate analyses of overall survival of the 91 patients indicated that the increased level in UTP11 is a poor prognostic factor in breast cancers (Fig. 7D and Supplementary Table 3). We also assessed the clinical relevance of UTP11 levels in colorectal cancer. Consistent with the results in breast cancer, UTP11 levels were higher in colorectal cancer tissues than in adjacent normal tissues, as determined by IHC staining (Fig. 7E and F). Also, higher levels of UTP11 were significantly associated with higher TNM stages and worse prognoses in a cohort of 150 patients with colorectal cancer (Fig. 7G and Supplementary Table 4). Furthermore, univariate and multivariate analyses revealed that UTP11 is a prognostic factor in colorectal cancer (Fig. 7H and Supplementary Table 5). Next, we explored the potential involvement of rRNA production and ROS levels in the prognoses of both cancers by employing MPP10 and SLC7A11 as markers, respectively. MPP10 mRNA (Supplementary Figs. 6A and 6B) and protein (Supplementary Figs. 6C and 6D) levels were elevated in breast and colorectal cancers compared with normal tissues. Higher levels of MPP10 were associated with worse survival in these two cancers (Supplementary Figs. 6E and 6F). Also, SLC7A11 expression was upregulated in breast and colorectal cancers (Supplementary Figs. 6G and 6H), and was associated with unfavorable prognoses (Supplementary Figs. 6I and 6J). Interestingly, UTP11 levels were positively correlated with the expression of both genes in cancer (Supplementary Fig. 6K-6N), suggesting that UTP11-mediated ribosome biogenesis and ferroptosis inhibition might contribute to a poor cancer prognosis. Taken together, our results suggest that UTP11 could be a biomarker for poor prognoses of both breast and colorectal cancers and a potential target for future development of a new anti-cancer therapy.

## 4 Discussion

Cancer cells sustain hyperactive ribosome biogenesis for the need of their rapid growth and proliferation. Growing evidence has shown that targeting components of this process can be a potential strategy for cancer therapy [7,8,45]. In this study, we identified UTP11 as a novel player in both the p53 and NRF-SLC7A11 pathways. Our RNA-seq analysis revealed that ablation of UTP11 activates several tumor suppressive pathways, including the p53 pathway and ferroptosis (Fig. 1B and C). Consistent with this result, UTP11 deficiency inhibits cancer growth *in vitro* and in vivo through both p53-dependent and independent mechanisms (Fig. 4, Fig. 5). Conversely, UTP11 overexpression promotes cancer cell growth and migration (Supplementary Fig. 4). On the one hand, UTP11 interacts with MPP10 to facilitate 18S rRNA synthesis; depletion of UTP11 induces RPL5/RPL11-dependent p53 activation via nucleolar stress (Fig. 2, Fig. 3). On the other hand, UTP11 stabilizes NRF2 mRNA by associating with the latter, whereas depleting UTP11 leads to the reduction in NRF2 mRNA's half-life and correspondingly the downregulation of its target gene *SLC7A11*, consequently leading to ferroptosis (Fig. 6; Supplementary Fig. 5). These results suggest an oncogenic role for UTP11. Indeed, UTP11 is often overexpressed in breast and colorectal cancers, and higher levels of UTP11 are associated with worse prognoses (Fig. 7). Taken together, our results demonstrate UTP11 as an oncoprotein that can promote cancer growth and proliferation by boosting ribosome biogenesis and suppressing ferroptosis involving p53-dependent and independent pathways.

Human UTP11 was originally identified through a comparative proteomic method using *Caenorhabditis elegans* proteome as a template [46]. However, its biological function has remained unclear for decades. In this study, we found that UTP11 is specifically required for biogenesis of 18S rRNA, but not 28S and 5.8/5S rRNAs (Fig. 2A–G). The perturbation of any step of ribosome biogenesis has been shown to activate the p53 pathway by triggering nucleolar stress [7,8]. For example, deficiency of large-subunit ribosomal proteins (RPLs) impairs the assembly of 60S ribosomal subunit [12,13,47], while depletion of small-subunit ribosomal proteins (RPSs) impairs the production of 40S ribosomal subunit [16,[48], [49], [50]]. Also, inhibition of some ribosomal factors, such as UTP17 and SBDS, induces nucleolar stress by impeding rRNA transcription and maturation [17,51]. We showed that the impairment of 18S rRNA synthesis caused by UTP11 depletion leads to nucleolar dysfunction, as evidenced by the translocation of the nucleolar marker, NPM1 (Fig. 2H). Mechanistically, UTP11 co-localized with and bound to MPP10 in the nucleolus, contributing to 18S rRNA biogenesis (Fig. 2I–L), which is consistent with the role of Utp11 in yeast [31]. These findings demonstrate for the first time that UTP11 plays an important role in ribosome biogenesis in mammalian cells.

Agents targeting the nucleolus have been developed to disrupt rRNA biogenesis and activate p53 as a strategy for cancer treatment. For instance, CX-3543 competes with NCL for binding to rDNA G-quadruplex structures [52]; CX-5461 prevents association of the SL1 transcription factor with rRNA gene promoter [53,54]; BMH-21 binds to GC-rich rDNA and impairs rRNA synthesis [55]. In addition, several traditional chemotherapeutic agents, which induce genotoxic damage, are believed to trigger nucleolar stress by impeding rDNA function or increasing rDNA damage [56]. The finding that UTP11 is critical for 18S rRNA generation strongly suggests that targeting this nucleolar protein may offer an alternative therapeutic strategy. Indeed, our results demonstrate that UTP11 knockdown mediates RPL5/RPL11-dependent p53 activation by enhancing the interactions between these two RPs and MDM2 (Fig. 3), which is a canonical mechanism of nucleolar stress-induced p53 activation [7,8]. UTP11 may also play a role in non-cancer diseases. It was reported that UTP11 is expressed in neurons of hippocampus, while downregulation of UTP11 may be associated with Alzheimer's disease, although the molecular basis is unclear [57]. Our study implies a potential mechanism for the causative role of UTP11 in neurodegenerative diseases, as nucleolar alterations and p53-induced cell death have been associated with the etiology of these diseases [58,59]. Thus, our study reveals a novel mechanism for UTP11 in cancer and possibly non-cancer diseases through the regulation of ribosome biogenesis and p53 activity.

Interestingly, we found that ablation of UTP11 also suppresses the growth of p53-null cancer cells (Fig. 5B, D, 5H, 5M − 5P). More interestingly, our study further demonstrated that targeting UTP11 promotes NRF2/SLC7A11-mediated ferroptosis. Several lines of evidence support this statement. First, our RNA-seq data indicated that UTP11 knockdown reduces the expression of SLC7A11 (Fig. 1C; Supplementary Fig. 5A), a component of the cystine/glutamate antiporter that is responsible for cystine uptake and GSH biogenesis [26,30]. Second, our IB and RT-qPCR analyses confirmed that UTP11 deficiency leads to the decline of SLC7A11 levels in a p53-independent manner (Fig. 6A–D; Supplementary Figs. 5B–5G). Third, the overexpression of UTP11 enhanced NRF2-mediated SLC7A11 transcription by increasing NRF2 mRNA stability (Fig. 6K–U; Supplementary Fig. 5S-5U). Finally, UTP11 knockdown reduced GSH levels to promote ferroptosis, while ectopic UTP11 increased GSH levels to inhibit ferroptosis independently of p53 (Fig. 6E–J; Supplementary Fig. 5L-5R). Notably, several other NRF2 target genes, such as HMOX1 and NQO1, which are associated with antioxidant activity and ferroptosis [43], were also downregulated in UTP11-deficient cancer cells (Fig. 6K, L, and 6 N; Supplementary Figs. 5S and 5U). It would be interesting to test whether these target genes are also involved in UTP11-regulated ferroptosis. Actually, there may be alternative mechanisms underlying UTP11 depletion-mediated inhibition of p53-null tumors, as nucleolar stress also elicits several RPs, such as RPL3, RPL11, and RPS14, to enhance oxidative stress and suppress cancer progression by repressing SLC7A11 expression or c-MYC activity [[60], [61], [62]]. Taken together, our results demonstrate that depleting UTP11 leads to the suppression of the antioxidant transcription factor NRF2 and consequently induces p53-independent ferroptosis.

In conclusion, our study as presented here identifies UTP11 as a new oncoprotein that acts to boost ribosome biogenesis and inhibit ferroptosis and is highly expressed and associated with poor prognoses in breast and colorectal cancers. Depletion of UTP11 promotes p53-dependent cell growth arrest via nucleolar stress and triggers ferroptosis via the NRF2-SLC7A11 axis. These findings suggest that UTP11 could be a potential therapeutic target for the treatment of cancer.

## Funding

This work was supported by the 10.13039/501100001809National Natural Science Foundation of China (82273098, 82072879, and 81874053 to X.Z., 82173022 to Q.H., 82171591 and 82060278 to J.C., 82260491 and 82202869 to J.D), 10.13039/501100012259Major Discipline Academic and Technical Leaders Training Program of Jiangxi Province (20204BCJ22031 to J.C.), the Jiangxi Provincial Young Talents projects (20204BCJ23016 to J.D.), the Key Laboratory of Jiangxi Province (20202BCD42011 to J.D.), the Henan Natural Science Fund for Excellent Young Scholars (212300410067 to T.H.), and in part by the Reynolds and Ryan Families Chair Fund of Translational Cancer to H.L.

## Author contributions

Y.G. and J.D. conducted most of the experiments and analyzed the data; Q.H. conducted part of experiments and analyzed the data; Y.H., T.H. and J.G.X analyzed the data; M.Z. performed bioinformatic analysis; L.Y., Y.X., and J.X. provided important experimental materials; H.L. provided helpful instruction; Q.H., C.W., J.C., and X.Z. conceived, designed and supervised the study and analyzed the data; H.L. and X.Z. wrote the paper.

## Availability of data and materials

The data generated or analyzed during this study are included in this published article.

## Ethics approval and consent to participate

The present study was approved by the ethics committee of the participating institutions.

## Declaration of competing interest

The authors declare no competing interests.
