# Supplementary material for: UTP11 deficiency suppresses cancer development via nucleolar stress and ferroptosis
Source: Redox Biol. 2023 Apr 17;62:102705. doi: 10.1016/j.redox.2023.102705 (PMC10149416; doi:10.1016/j.redox.2023.102705)

UTP11 deficiency suppresses cancer development via nucleolar stress and ferroptosis

Yu Gan[1,2,#], Jun Deng[3,#], Qian Hao[2,4], Yingdan Huang[2,4], Tao Han[5], Jin-Guo Xu[5], Min Zhao[6], Litong Yao[7], Yingying Xu[7], Jianping Xiong[3], Hua Lu[8], Chunmeng Wang[4,9,*], Jiaxiang Chen[1,*], and Xiang Zhou[2,4,10,11,*]

[1] Department of Physiology, School of Basic Medical Sciences, Nanchang University, Nanchang 330006, China.

[2] Fudan University Shanghai Cancer Center and Institutes of Biomedical Sciences, Fudan University, Shanghai 200032, China.

[3] Department of Oncology, The First Affiliated Hospital of Nanchang University, Nanchang 330006, China.

[4] Department of Oncology, Shanghai Medical College, Fudan University, Shanghai 200032, China.

[5] Institutes of Health Central Plains, Xinxiang Medical University, Xinxiang 453003, China.

[6] School of Science, Technology and Engineering, University of the Sunshine Coast, Maroochydore DC, Queensland 4558, Australia.

[7] Department of Breast Surgery, the First Affiliated Hospital of China Medical University, Shenyang 110001, China.

[8] Department of Biochemistry & Molecular Biology and Tulane Cancer Center, Tulane University School of Medicine, New Orleans, LA 70112, USA.

[9] Department of Musculoskeletal Oncology, Fudan University Shanghai Cancer Center, Shanghai 200032, China.

[10] Key Laboratory of Breast Cancer in Shanghai, Fudan University Shanghai Cancer Center, Fudan University, Shanghai, 200032, China.

[11] Shanghai Key Laboratory of Medical Epigenetics, International Co-laboratory of Medical Epigenetics and Metabolism (Ministry of Science and Technology), Institutes of Biomedical Sciences, Fudan University, Shanghai 200032, China.

[#] Equal contribution

[*] Correspondence:

Xiang Zhou, Email: xiangzhou@fudan.edu.cn

Jiaxiang Chen, Email: chenjiaxiang@ncu.edu.cn

Chunmeng Wang, Email: cmwang1975@163.com

Supplementary Figure legends

Supplementary Figure 1. Identification of UTP11 as a potential oncoprotein in cancer. (A) Analysis of protein-protein interacting network between 27 UTP proteins and the p53 signaling proteins reveals that 11 UTP proteins, including UTP11, are directly or indirectly associated with the p53 pathway. **(B)** Eight of the 11 UTP proteins have been reported to be involved in the regulation of the p53 signaling pathway or cell growth. **(C)** UTP11 is highly associated with the poor prognosis in breast cancer by the Kaplan–Meier survival analysis.

Supplementary Figure 2. Clinical relevance of UTP11 levels in various cancers. (A) The *UTP11* gene is amplified in several human cancers from TCGA database. **(B)** UTP11 mRNA levels are upregulated in various human cancers from TCGA database. **(C-F)** UTP11 protein levels are higher in several human cancers, including breast cancer (C), colon cancer (D), hepatocellular carcinoma (E), and lung adenocarcinoma (F), than normal tissues. **(G-K)** Higher expression of UTP11 is correlated with worse prognoses in different human cancers, including esophageal adenocarcinoma (G), hepatocellular carcinoma (H), kidney renal papillary cell carcinoma (I), rectum adenocarcinoma (J), and uterine corpus endometrial carcinoma (K).

Supplementary Figure 3. UTP11 overexpression inhibits p53 activity at protein level. (A-C) Ablation of UTP11 has no effect on p53 mRNA levels. CAL-51 (A), MCF-7 (B), and HCT116 $^{p53+/+}$ cells (C) were transfected with control or UTP11 siRNA, followed by RT-qPCR analysis. **(D-G)** Overexpression of UTP11 reduces the expression of p53 and p21. CAL-51 (A, B) and HCT116 $^{p53+/+}$ cells (C, D) were transfected with control or UTP11 siRNAs, followed by IB and RT-qPCR analyses. *** $p<0.001$.

Supplementary Figure 4. UTP11 overexpression promotes breast and colorectal cancer cell growth and migration. (A, B) CAL-51 (A) and HCT116 $^{p53+/+}$ (B) cells were infected with lentivirus encoding PCDH or PCDH-UTP11, followed by IB analysis. **(C, D)** Overexpression of UTP11 promotes cancer cell proliferation. The above CAL-51 (C) and HCT116 $^{p53+/+}$ (D) cells were subjected to cell viability assay. **(E, F)** Overexpression of UTP11 enhances the colony-forming ability of cancer cells. The above CAL-51 (E) and HCT116 $^{p53+/+}$ (F) cells were subjected to colony formation assay. **(G, H)** Overexpression of UTP11 increases the migration of cancer cells. The above CAL-51 (G) and HCT116 $^{p53+/+}$ (H) cells were subjected to transwell cell migration assay. * $p<0.05$, ** $p<0.01$, *** $p<0.001$.

Supplementary Figure 5. UTP11 regulates the NRF2-SLC7A11 axis. (A) RNA-sequencing results reveal that a panel of ferroptosis-related genes are markedly altered in UTP11-depleted cells. **(B-G)** Knockdown of UTP11 leads to the reduction of SLC7A11 mRNA and protein levels. CAL-51 (B, C), MCF-7 (D, E), and RKO cells (F, G) transfected with control or UTP11

siRNAs were subjected to RT-qPCR and IB analyses with indicated antibodies. **(H-K)** Overexpression of UTP11 elevates both mRNA and protein levels of SLC7A11 and NRF2 independently of p53. HCT116 $^{p53+/+}$ (H, I) and HCT116 $^{p53-/-}$ cells (J, K) stably expressing the indicated plasmids were subjected to RT-qPCR and IB analyses. **(L)** UTP11 depletion reduces GSH level. CAL-51 cells were transfected with control or UTP11 siRNAs, followed by GSH assay. **(M, N)** UTP11 overexpression increases GSH levels. HCT116 $^{p53+/+}$ (M) and HCT116 $^{p53-/-}$ cells (N) stably expressing the indicated plasmids were subjected to GSH assay. **(O)** UTP11 depletion increases MDA level. CAL-51 cells were transfected with control or UTP11 siRNAs, followed by MDA assay. **(P, Q)** UTP11 overexpression decreases MDA levels. HCT116 $^{p53+/+}$ (P) and HCT116 $^{p53-/-}$ cells (Q) stably expressing the indicated plasmids were subjected to MDA assay. **(R)** UTP11 depletion-mediated cell growth inhibition is partially restored by Ferrostatin-1 treatment. CAL-51 cells after being transfected with the indicated siRNAs were seeded in 96-well plates and treated with DMSO or Ferrostatin-1 (2 μM) for 48 h. Cells were harvested for cell viability assay. **(S-U)** Knockdown of UTP11 represses the expression of NRF2 and its target genes. CAL-51 (S, T) and RKO (U) cells were transfected with control or UTP11 siRNAs, followed by RT-qPCR and IB analyses with indicated antibodies. * $p<0.05$, ** $p<0.01$, *** $p<0.001$.

Supplementary Figure 6. Clinical relevance of MPP10 and SLC7A11 levels in breast and colon cancers. (A, B) MPP10 mRNA levels are upregulated in breast (A) and colon (B) cancers from TCGA database. **(C, D)** MPP10 protein levels are elevated in breast (C) and colon (D) cancers from CPTAC database. **(E, F)** Higher expression levels of MPP10 are associated with worse prognoses in breast (E) and colon (F) cancers. **(G, H)** SLC7A11 mRNA levels are upregulated in breast (G) and colon (H) cancers from TCGA database. **(I, J)** Higher expression levels of SLC7A11 are associated with worse prognoses in breast (I) and colon (J) cancers. **(K, L)** The expression of MPP10 is positively correlated with UTP11 levels in breast (K) and colon (L) cancers. **(M, N)** The expression of SLC7A11 is positively correlated with UTP11 levels in breast (M) and colon (N) cancers.

Supplementary Figure 1

A

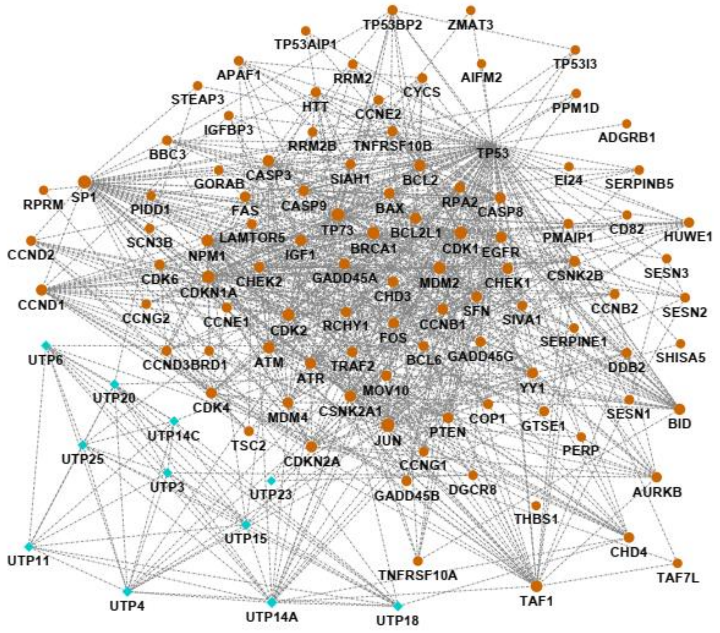

B

Gene	Relative literatures in PubMed
UTP3	PMID: 36245126
UTP4	PMID: 24147052
UTP14(UTP14A)	PMID: 21078665; PMID: 30343112; PMID: 33391409
UTP15	PMID: 21949834
UTP18	PMID: 25435373
UTP20	PMID: 21151873
UTP23	PMID: 31540773
UTP25	PMID: 34452850

C

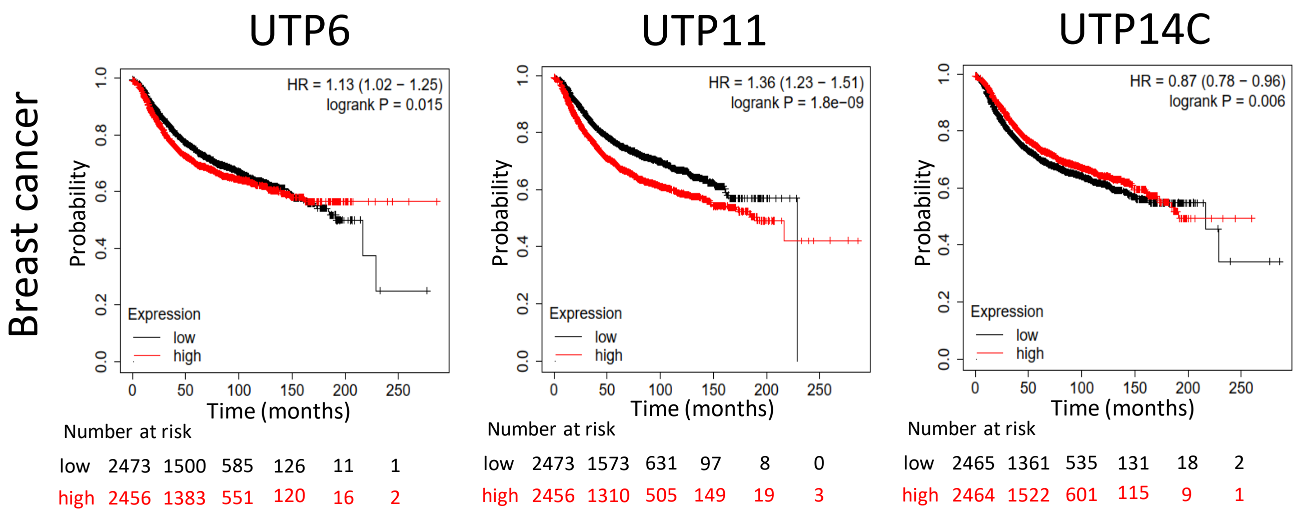

Supplementary Figure 2

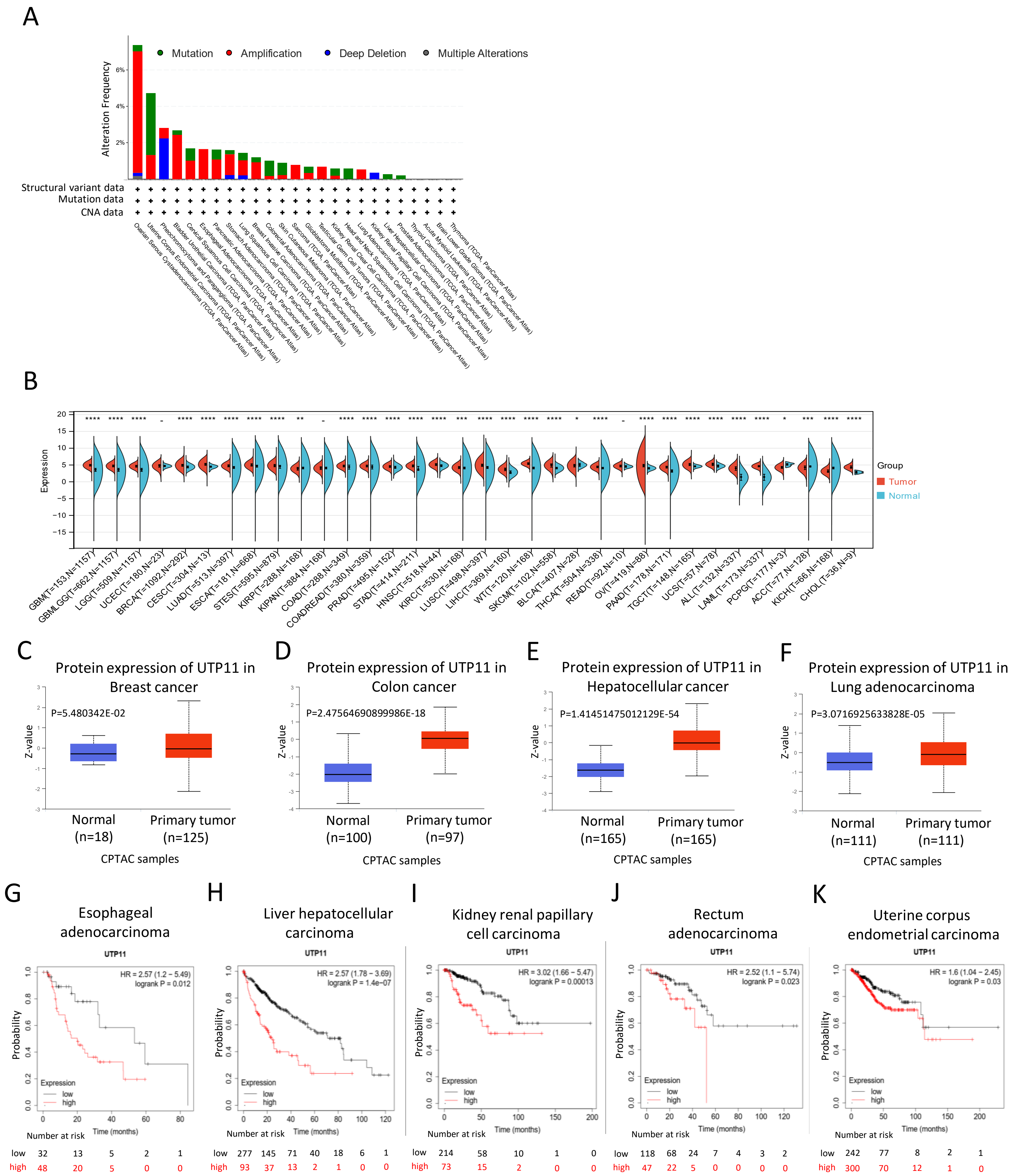

Supplementary Figure 3

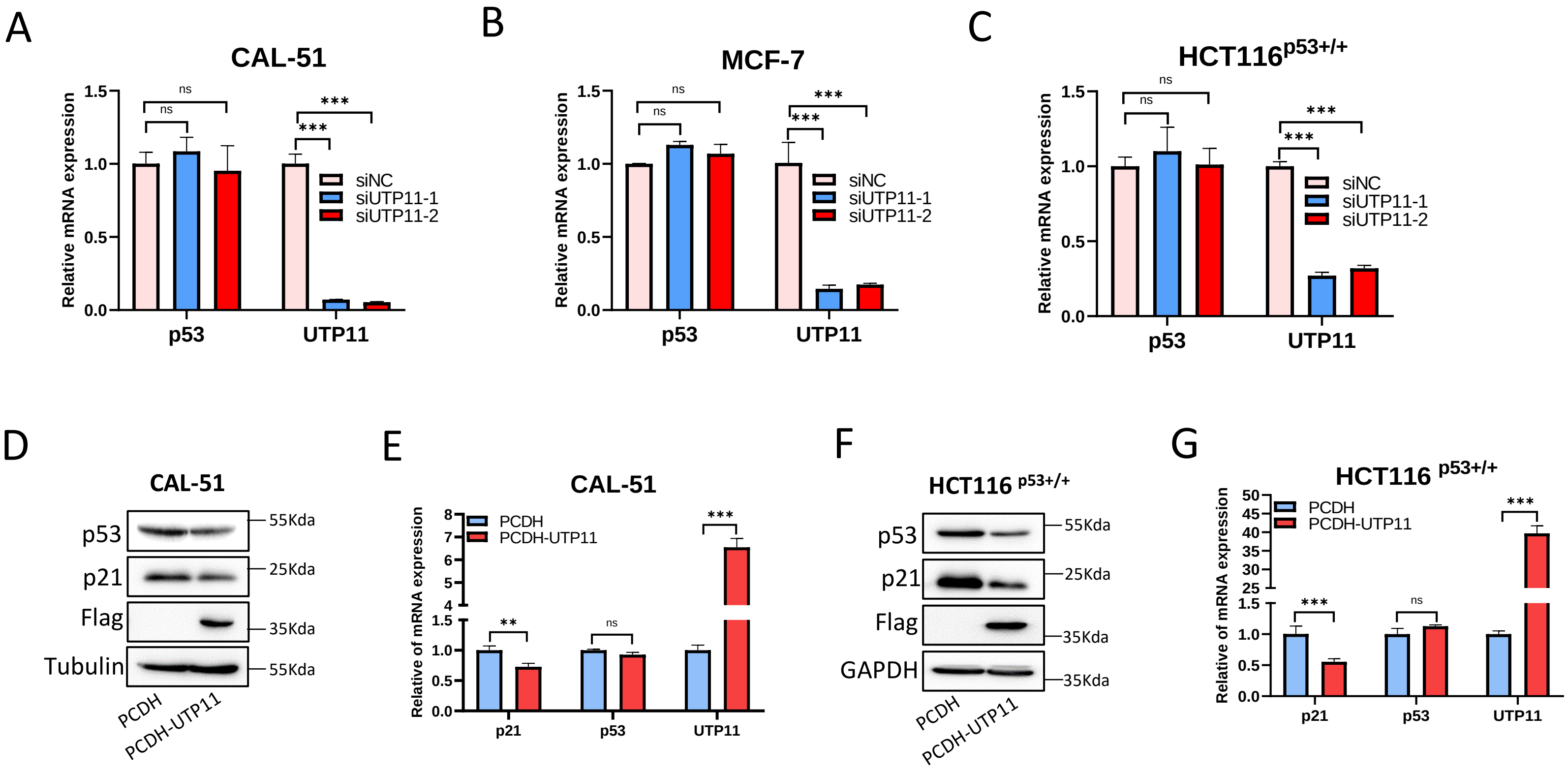

Supplementary Figure 4

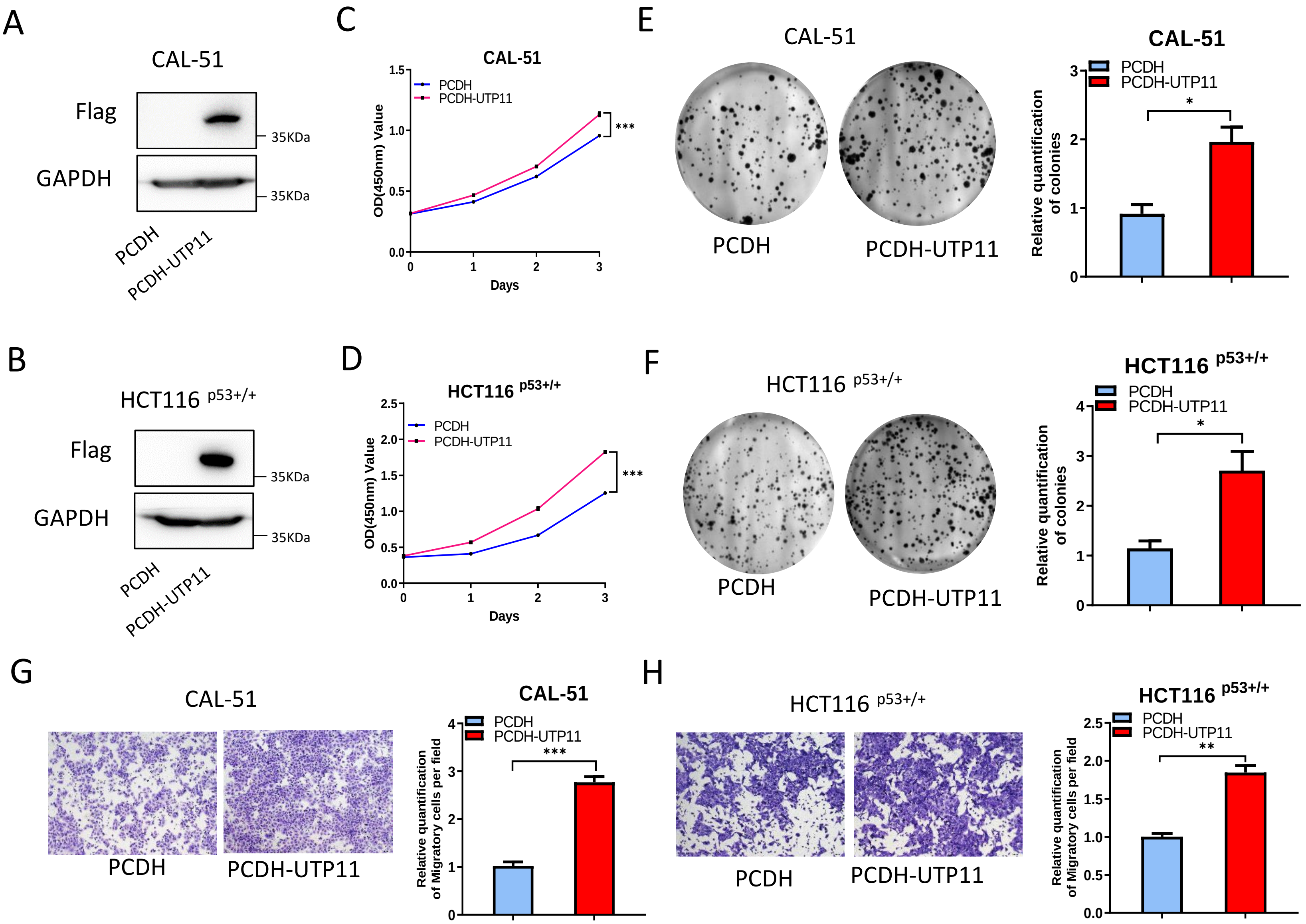

Supplementary Figure 5

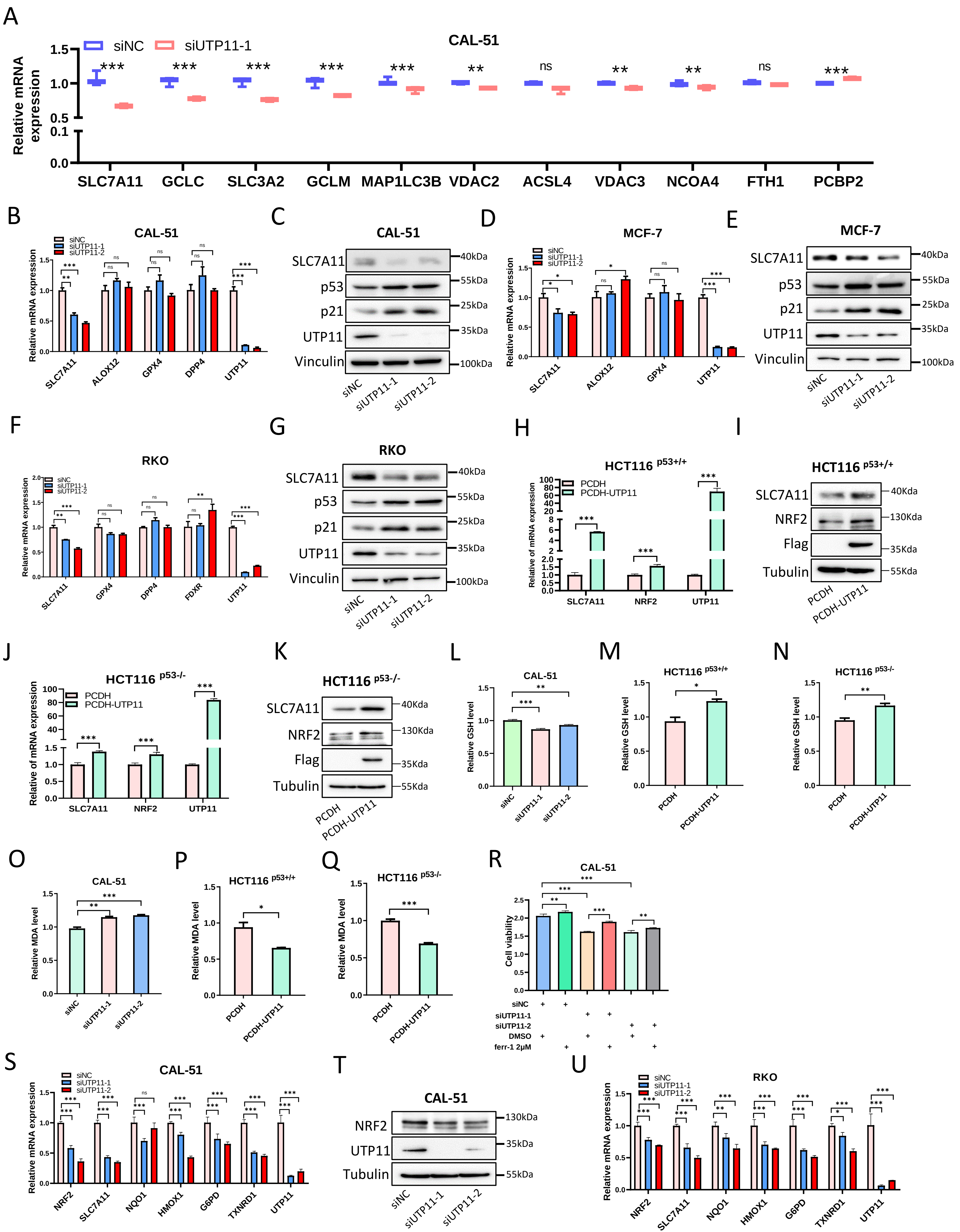

Supplementary Figure 6

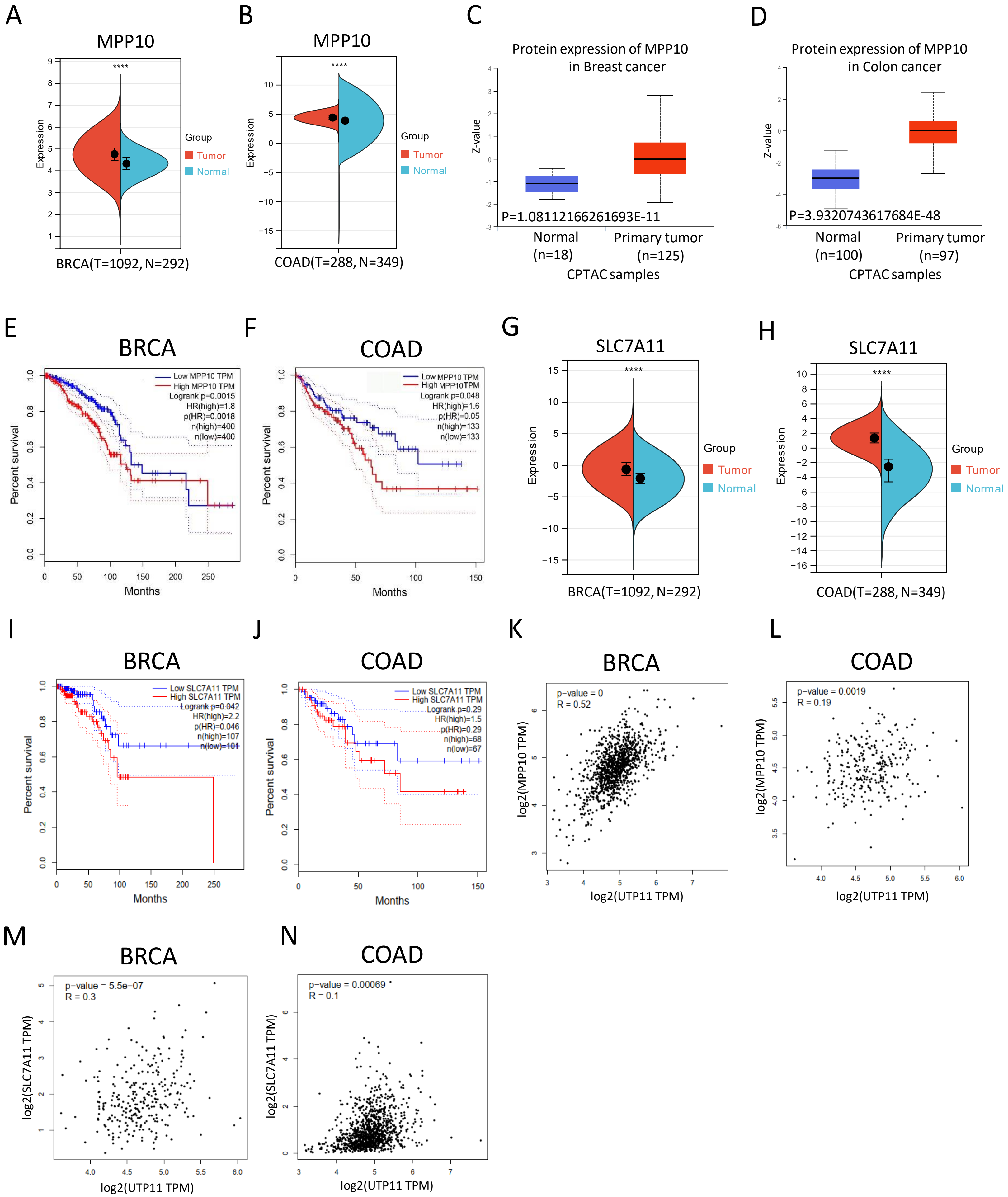

Supplement: Multimedia component 1 [file mmc1.pdf]
