# Supplementary material for: UTP11 deficiency suppresses cancer development via nucleolar stress and ferroptosis
Source: Redox Biol. 2023 Apr 17;62:102705. doi: 10.1016/j.redox.2023.102705 (PMC10149416; doi:10.1016/j.redox.2023.102705)
Supplement: Multimedia component 3 [file mmc3.docx]

**Supplementary Tables**

**Supplementary Tables 2:** Relationship between UTP11 expression and clinicopathologic factors of patients with breast cancer.

| **Variables** | **High（n=26）** | **%** | **Low（n=65）** | **%** |  | **p-value** |
| --- | --- | --- | --- | --- | --- | --- |
| Age（years） |  |  |  |  |  |  |
| ≥50 | 17 | 29.8 | 40 | 70.2 |  | 0.732 |
| <50 | 9 | 26.5 | 25 | 73.5 |  |  |
| Tumor size |  |  |  |  |  |  |
| ≥2cm | 19 | 28.4 | 48 | 71.6 |  | 0.940 |
| <2cm | 7 | 29.2 | 17 | 70.8 |  |  |
| Lymph node |  |  |  |  |  |  |
| N1-N3 | 18 | 40.0 | 27 | 60.0 |  | **0.014** |
| N0 | 8 | 17.4 | 38 | 82.6 |  |  |

Comparison was determined by Chi-square test or Fisher's exact test.

**Supplementary Tables 3:** Multivariate cox regression analysis of OS in 91 BRCA.

| Variables | Univariate analysis | | Multivariate analysis  (forward stepwise) | |
| --- | --- | --- | --- | --- |
|  | HR (95%CI) | p value | HR (95%CI) | P value |
| UTP11 (high vs. low) | 4.160  (1.867-9.272) | **0.001** | 3.397  (1.481-7.792) | **0.004** |
| Age(years) (≥60 vs. <60) | 2.998  (1.123-8.007) | **0.028** | 3.457  (1.270-9.045) | **0.015** |
| Tumor size  (≥2cm vs. <2cm) | 0.894  (0.372-2.152) | 0.803 | 0.976  (0.397-2.403) | 0.985 |
| Lymph node  (N1-3 vs. N0) | 3.128  (1.331-7.348) | **0.009** | 2.589  (1.068-6.276) | **0.035** |

Factors predicting OS were analyzed by univariate Cox’s proportional hazard regression models. OS: overall survival; BRCA: breast cancer; HR: hazard ratio; CI: confidence intervals.

**Supplementary Tables 4:** Relationship between UTP11 expression and clinicopathologic factors of patients with colorectal cancer.

| **UTP11 Expression Level** | | | | | |
| --- | --- | --- | --- | --- | --- |
| **Variables** | **High（n=85）** | **%** | **Low（n=65）** | **%** | **p value** |
| Age（years） |  |  |  |  | 0.2520 |
| <60 | 40 | 51.95 | 37 | 48.05 |  |
| ≥60 | 45 | 61.64 | 28 | 38.36 |  |
| Gender |  |  |  |  | 0.1232 |
| male | 50 | 62.50 | 30 | 37.50 |  |
| female | 35 | 50.00 | 35 | 50.00 |  |
| Location |  |  |  |  | 0.5704 |
| colon | 51 | 58.62 | 36 | 41.38 |  |
| rectum | 34 | 53.97 | 29 | 46.03 |  |
| Differentiation |  |  |  |  | 0.7355 |
| moderate or high | 61 | 57.55 | 45 | 42.45 |  |
| low | 24 | 54.55 | 20 | 45.45 |  |
| TNM stage |  |  |  |  | **0.0038** |
| I+II | 36 | 45.57 | 43 | 54.43 |  |
| III+IV | 49 | 69.01 | 22 | 30.99 |  |

Comparison was determined by Chi-square test or Fisher's exact test.

**Supplementary Tables 5:** Multivariate cox regression analysis of OS in 150 CRC.

| Variables | Univariate analysis | | Multivariate analysis (forward stepwise) | |
| --- | --- | --- | --- | --- |
|  | HR (95%CI) | p value | HR (95%CI) | p value |
| Age(years) (<60 vs. ≥60) | 1.482  (0.933-2.354) | 0.095 | - | - |
| Gender (male vs. female) | 0.689  (0.437-1.084) | 0.107 | - | - |
| Differentiation (moderate or high vs. low) | 0.726  (0.449-1.173) | 0.191 | - | **-** |
| Location  (colon vs. rectum) | 1.547  (0.961-2.492) | 0.072 | - | - |
| TNM stage  (I + II vs. III+IV) | 0.400  (0.251-0.637) | **p<0.001** | 0.439  (0.273-0.704） | **0.001** |
| UTP11 (high vs. low) | 1.500  (1.309-1.810) | **0.005** | 1.577  (1.354-1.941） | **0.028** |

Factors predicting OS were analyzed by univariate Cox’s proportional hazard regression models. The factors with P < 0.05 in univariate Cox’s regression were further analyzed in forward stepwise multivariate Cox’s regression. OS: overall survival; CRC: colorectal cancer; HR: hazard ratio; CI: confidence interval.

**Supplementary Table 6.** A list of primers for RT-qPCR

| GAPDH-F | 5’-GGAGCGAGATCCCTCCAAAAT-3’ |
| --- | --- |
| GAPDH-R | 5’-GGCTGTTGTCATACTTCTCATGG-3’ |
| p21-F | 5’-CTGGACTGTTTTCTCTCGGCTC-3’ |
| p21-R | 5’-TGTATATTCAGCATTGTGGGAGGA-3’ |
| BTG2-F | 5’-ACGGGAAGGGAACCGACAT-3’ |
| BTG2-R | 5’-CAGTGGTGTTTGTAGTGCTCTG-3’ |
| MDM2-F | 5’-GAATCATCGGACTCAGGTACATC-3’ |
| MDM2-R | 5’-TCTGTCTCACTAATTGCTCTCCT-3’ |
| UTP11-F | 5’-ATACGGGATCCATGGCGGCGGCTTTTC-3’ |
| UTP11-R | 5’-CCGCTCGAGTCAACGTTTTCGACGACTCT-3’ |
| P53-F | 5’-CCCAAGCAATGGATGATTTGA-3 |
| P53-R | 5’-GGCATTCTGGGAGCTTCATCT-3 |
| 28S-F | 5’-AGAGGTCTTGGGGCCGAAACGATCTCAACC-3’ |
| 28S-R | 5’-CTGATGAGCGTCGGCATCGGGCGCCTTAAC-3’ |
| 18S-1F | 5′-CGCTTCCTTACCTGGTTGAT-3′ |
| 18S-1R | 5’-GCCCGAGGTTATCTAGAGTCACCAAAGCCG-3’ |
| 18S-2F | 5′-CGCTTCCTTACCTGGTTGAT-3′ |
| 18S-2R | 5′-GAGCGACCAA AGGAACCATA-3′ |
| 5S-F | 5’- GGCCATACCACCCTGAACGC-3’ |
| 5S-R | 5’- CAGCACCCGGTATTCCCAGG-3’ |
| SLC7A11-F | 5’-TCTCCAAAGGAGGTTACCTGC-3’ |
| SLC7A11-R | 5’-AGACTCCCCTCAGTAAAGTGAC-3’ |
| GPX4-F | 5’-TTCCCGTGTAACCAGTTCG-3’ |
| GPX4-R | 5’-CGGCGAACTCTTTGATCTCT-3’ |
| DPP4-F | 5’-AAAGGCACCTGGGAAGTCATCG-3’ |
| DPP4-R | 5’-CAGCTCACAACTGAGGCATGTC-3’ |
| FDXR-F | 5’-TGGAAATTCCTGGTGAGGAG-3’ |
| FDXR-R | 5’-CTGGAGACCCAAGAAATCCA-3’ |
| ALOX12-F | 5’-GCTCCTGGAACTGCCTAGAA-3’ |
| ALOX12-R | 5’-TCATCATCCTGCCAGCACT-3’ |
| NRF2-F | 5’-TCAGCGACGGAAAGAGTATGA-3’ |
| NRF2-R | 5’-CCACTGGTTTCTGACTGGATGT-3’ |
| NQO1-F | 5’-GAAGAGCACTGATCGTACTGGC-3’ |
| NQO1-R | 5’-GGATACTGAAAGTTCGCAGGG-3’ |
| HMOX1-F | 5’-AAGACTGCGTTCCTGCTCAAC-3’ |
| HMOX1-R | 5’-AAAGCCCTACAGCAACTGTCG-3’ |
| G6PD-F | 5’-CGAGGCCGTCACCAAGAAC-3’ |
| G6PD-R | 5’-GTAGTGGTCGATGCGGTAGA-3’ |
| TXNRD1-F | 5’-ATATGGCAAGAAGGTGATGGTCC-3’ |
| TXNRD1-R | 5’-GGGCTTGTCCTAACAAAGCTG-3’ |

**Supplementary Table 7.** A list of primers for plasmid construction

| MPP10-XbaI-Myc-his-PCDNA3.1-F | 5’- GCTCTAGAATGGCGCCGCAGGT -3’ |
| --- | --- |
| MPP10-EcoRI-Myc-his-PCDNA3.1-R | 5’CGGAATTCGCAGCTTTAATTTATGAACAGAAATATCC -3’ |
| UTP11-XhoI-flag PCDNA3.1-F | 5’- ATACGGGATCCATGGCGGCGGCTTTTC -3’ |
| UTP11-BamHI-flag PCDNA3.1-R | 5’- CCGCTCGAGTCAACGTTTTCGACGACTCT -3’ |

**Supplementary Table 8.** A list of primers for RIP-qPCR

| NRF2-1-F | 5’-TGCCAACTACTCCCAGGTTG-3’ |
| --- | --- |
| NRF2-1-R | 5’-AGACTGGGCTCTCGATGTG-3’ |
| NRF2-2-F | 5’-GCCCTCACCTGCTACTTTAAGC-3’ |
| NRF2-2-R | 5’-TCACTGAGGCCAAGTAGTGTGTC-3’ |
