# Supplementary material for: UTP11 deficiency suppresses cancer development via nucleolar stress and ferroptosis
Source: Redox Biol. 2023 Apr 17;62:102705. doi: 10.1016/j.redox.2023.102705 (PMC10149416; doi:10.1016/j.redox.2023.102705)

Figure1D

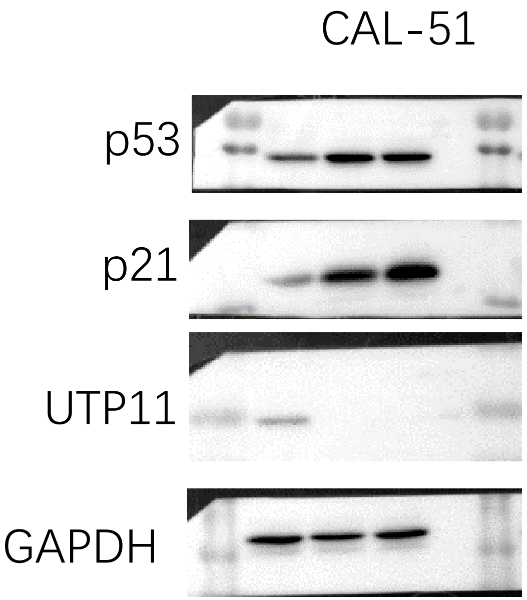

Figure1F

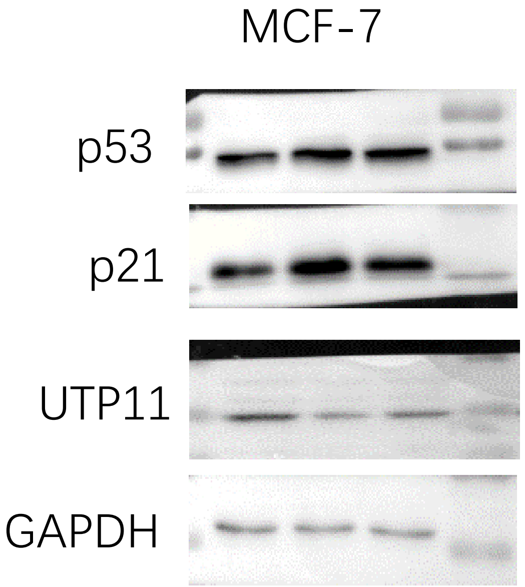

Figure1H

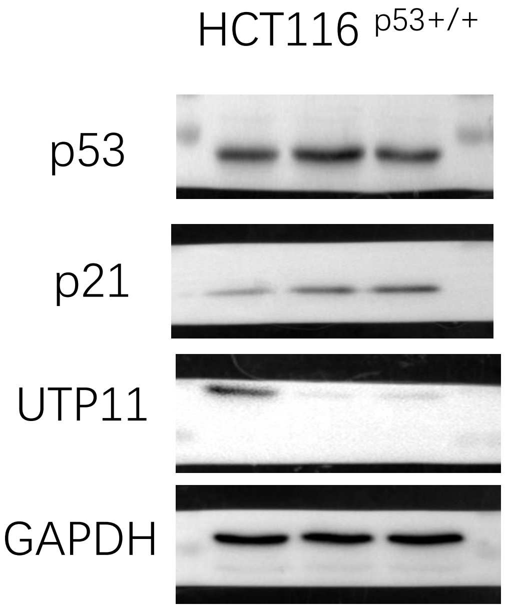

Figure1J

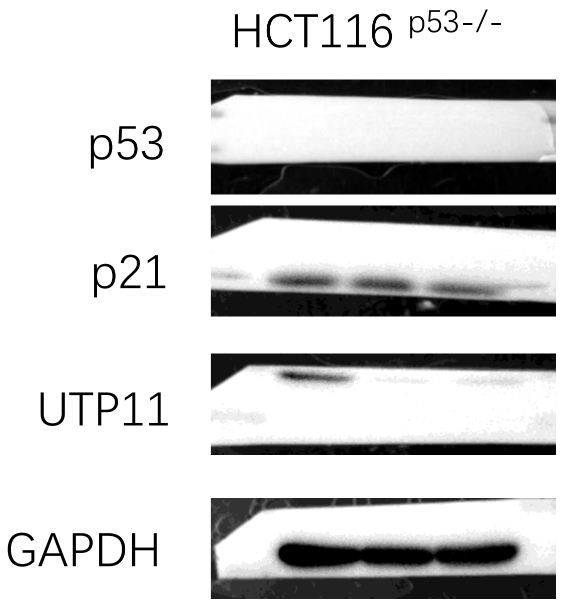

Figure2J

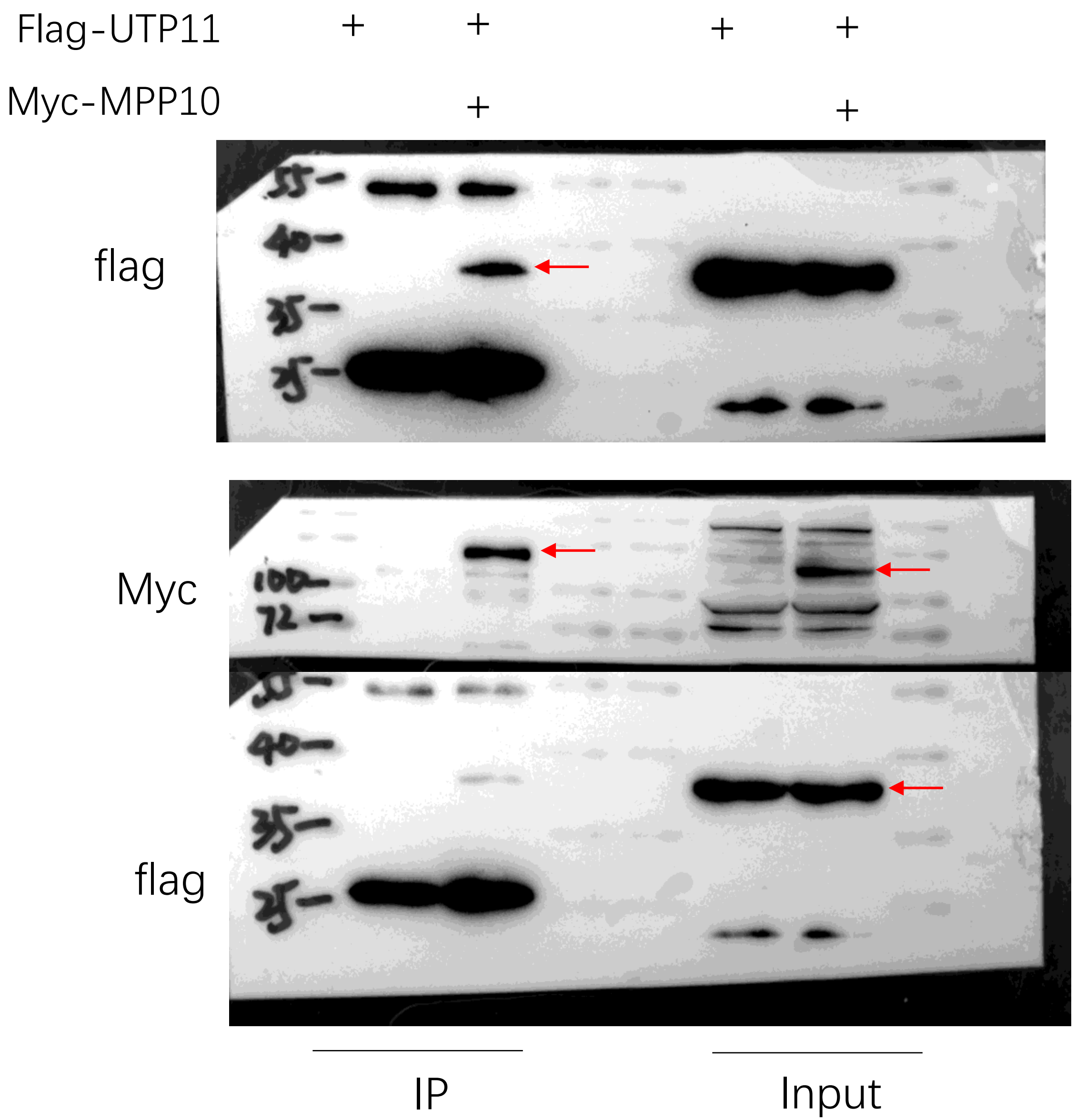

Figure2K

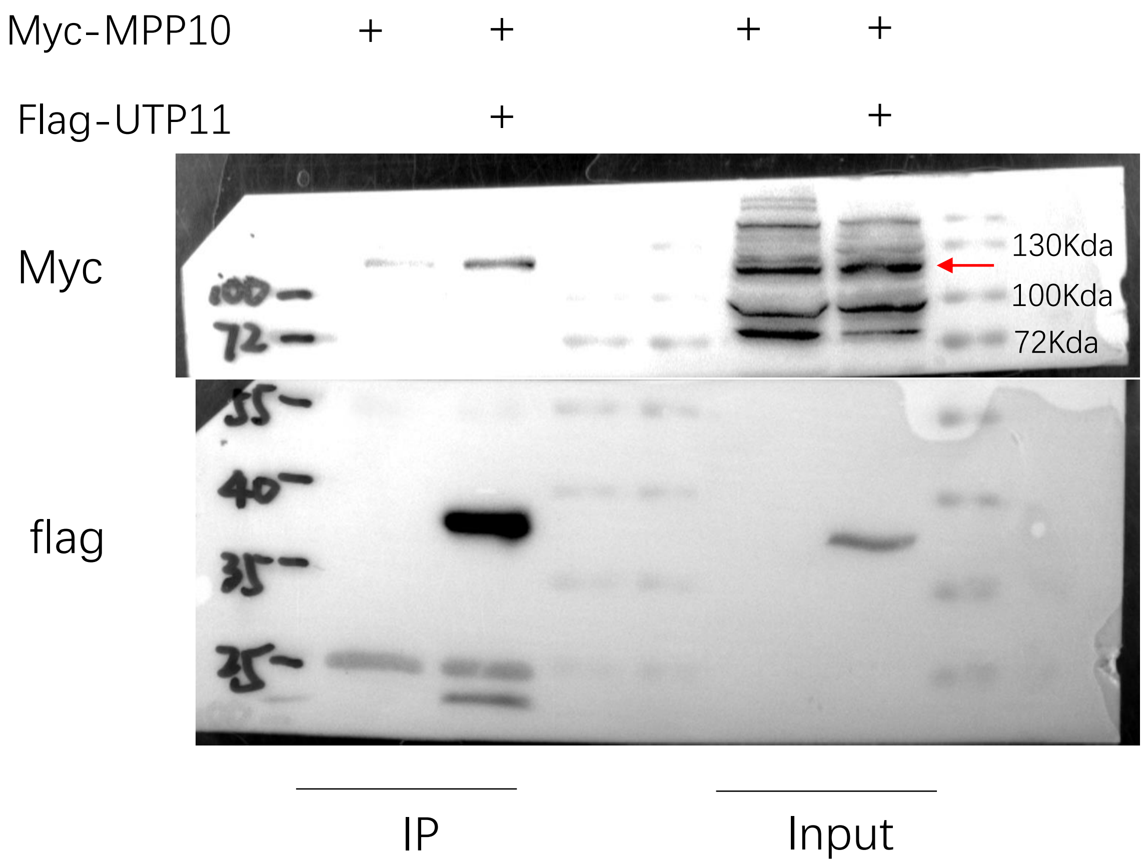

Figure 2L

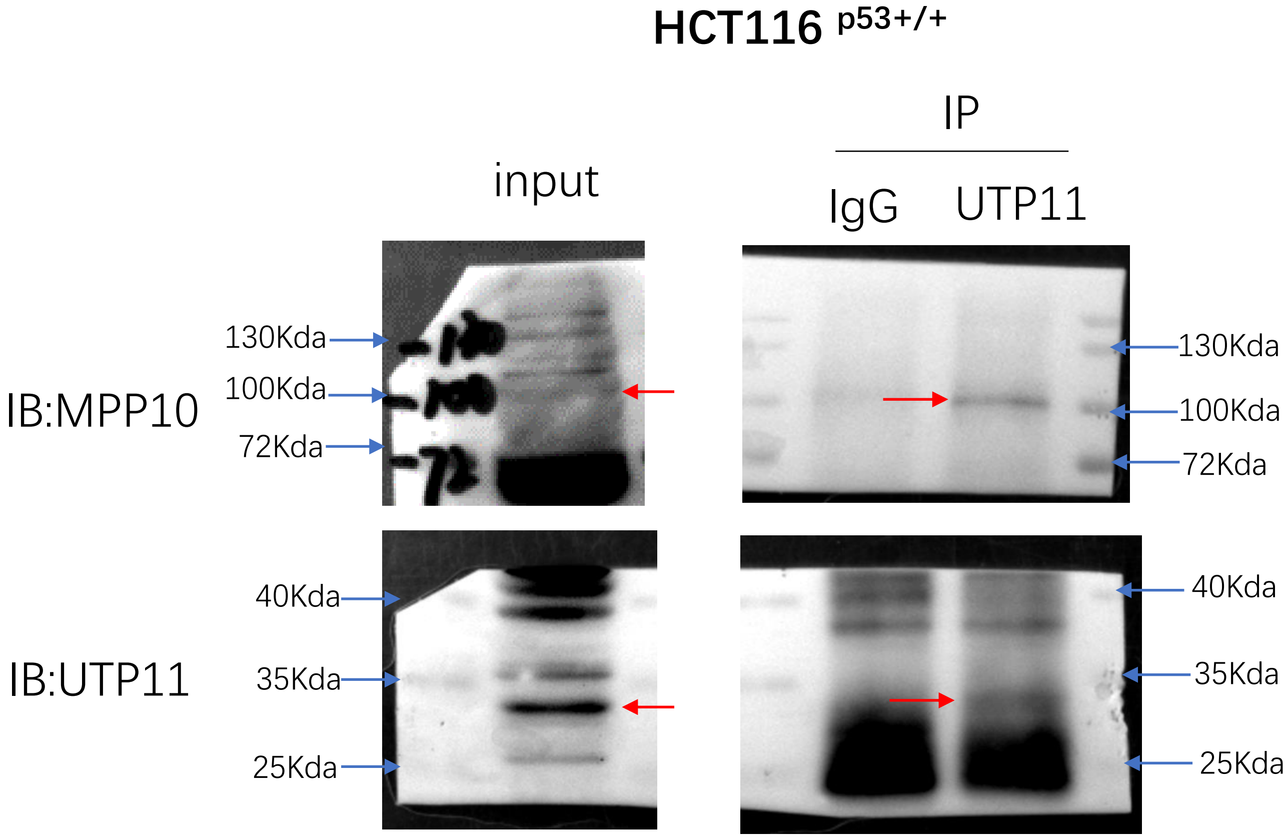
HCT116 p53+/+
IP
input
IgG
UTP11
IB:MPP10
130Kda
100Kda
72Kda
130Kda
100Kda
72Kda
IB:UTP11
40Kda
35Kda
25Kda
40Kda
35Kda
25Kda

Figure 3A

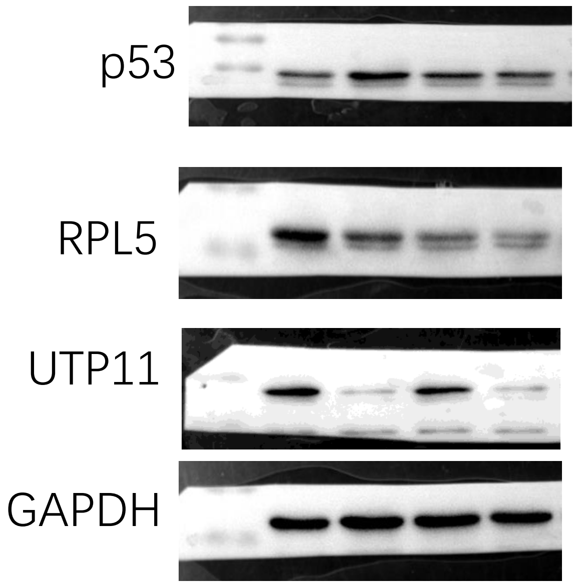

Figure 3B

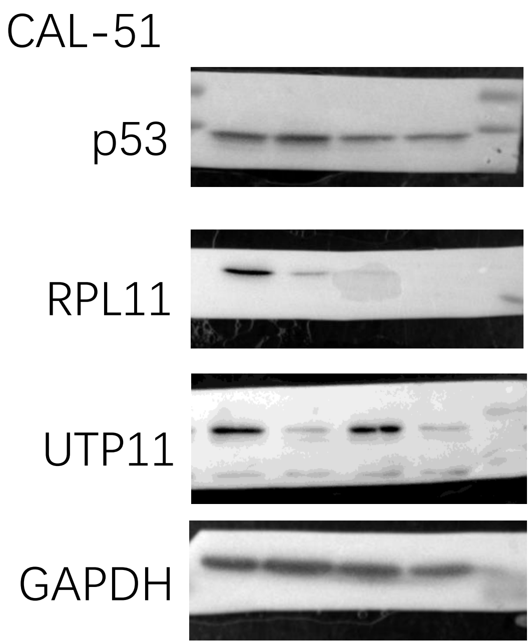

Figure 3C

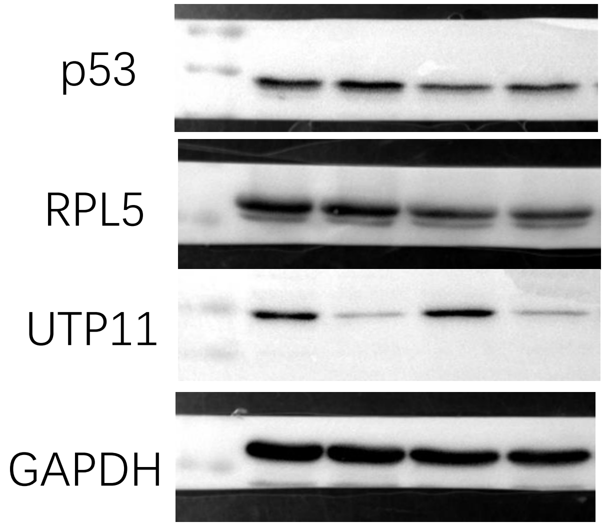

Figure 3D

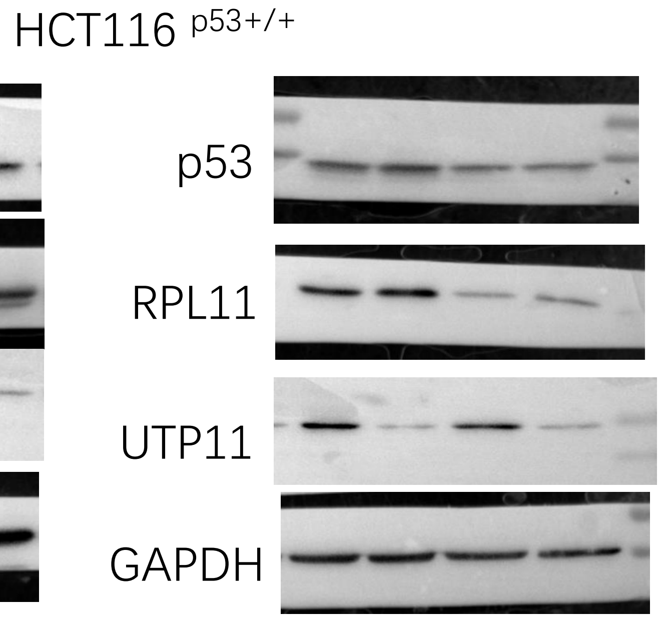

CAL-51

Figure 3E

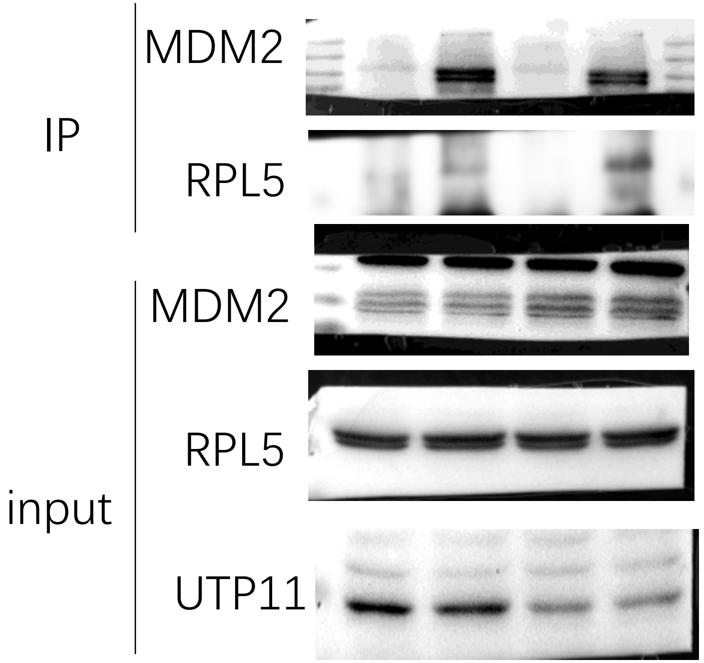

Figure 3F

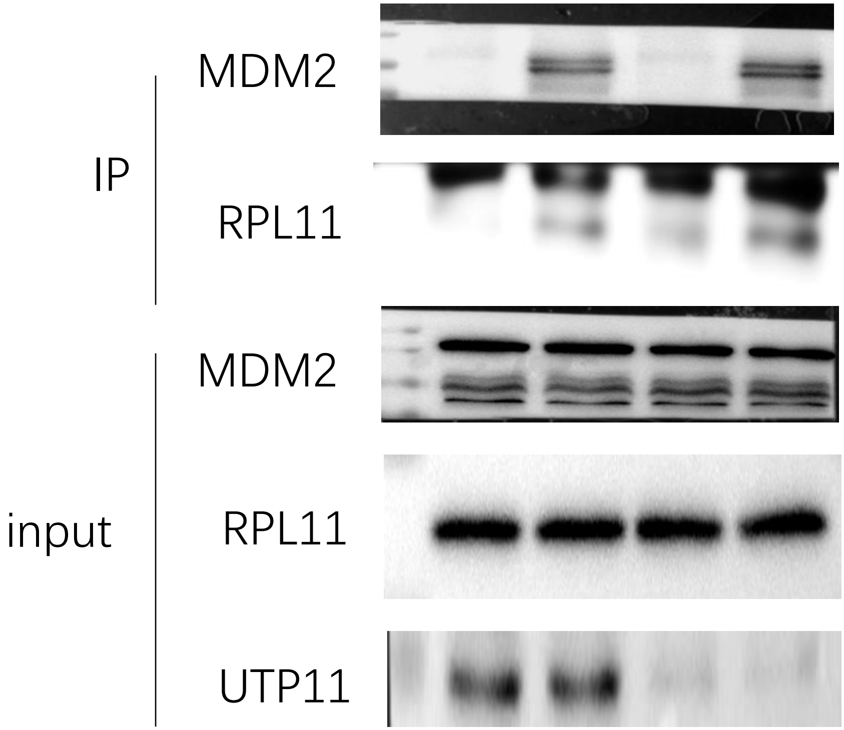

Figure 3G

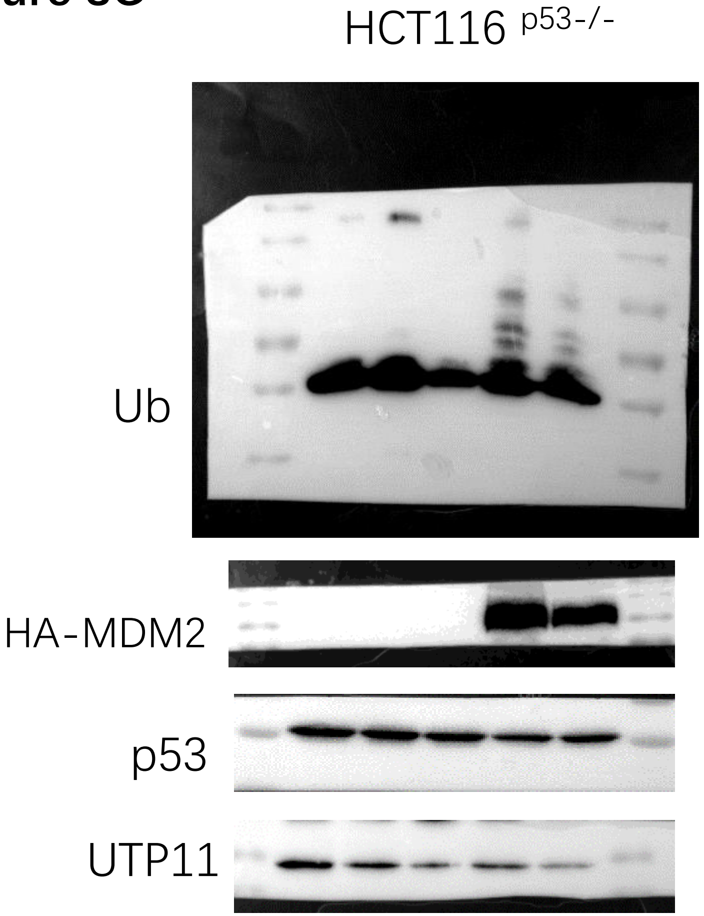

Figure 3H

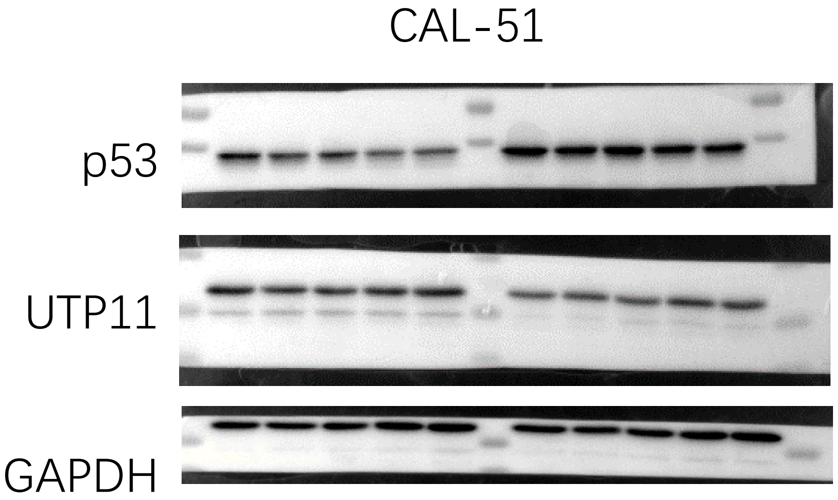

Figure 6B

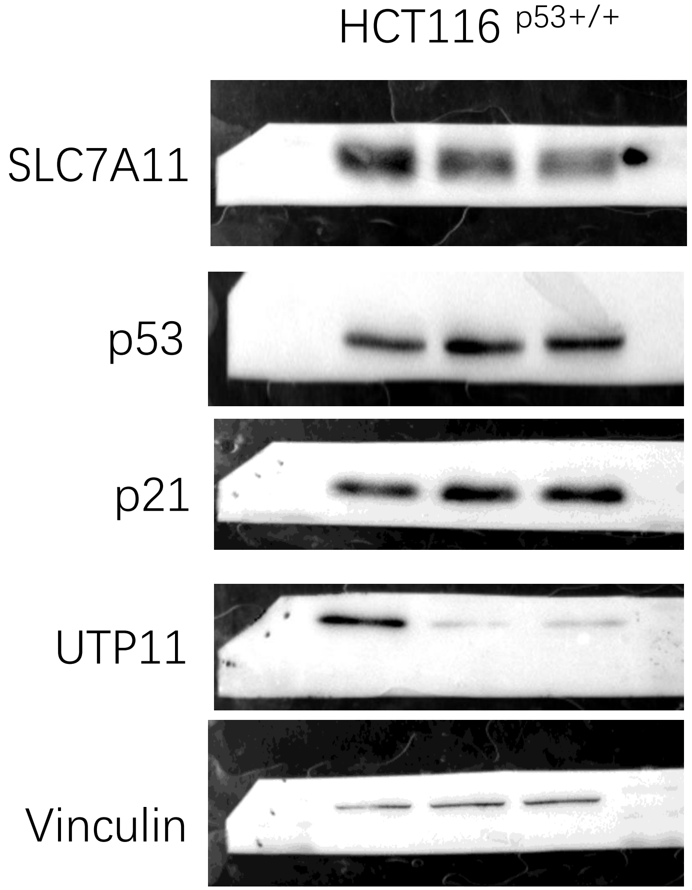

Figure 6D

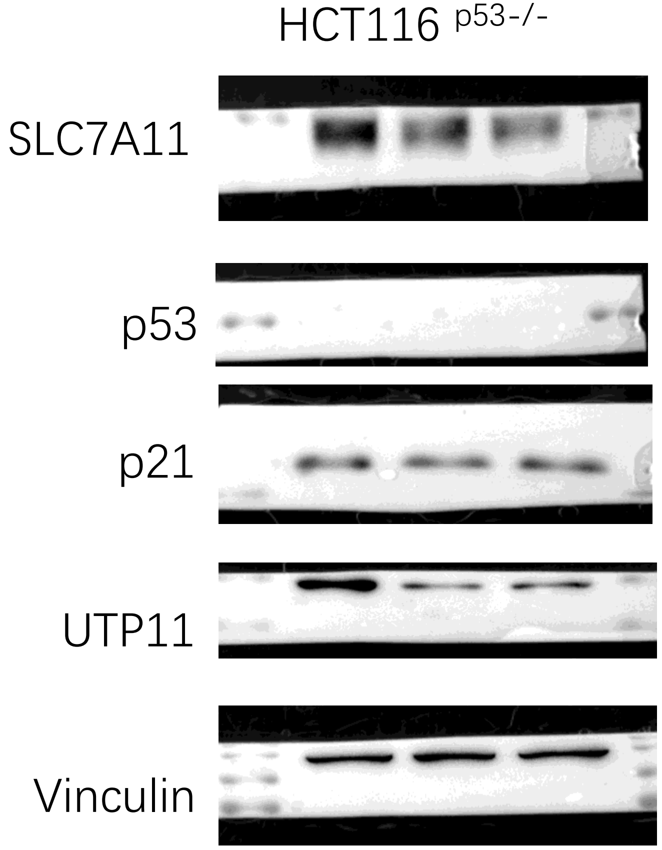

Figure 6M

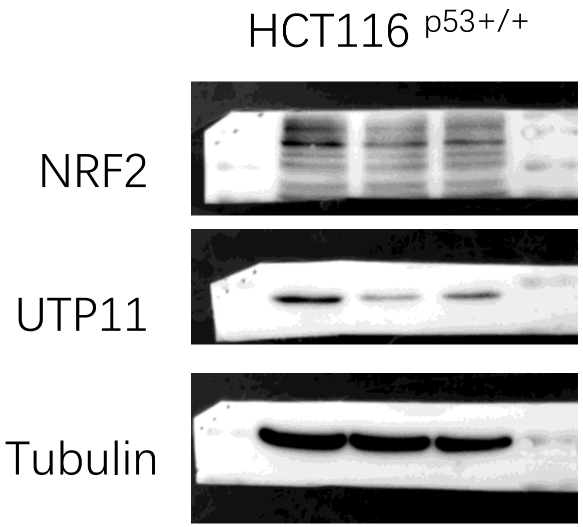

Figure 6O

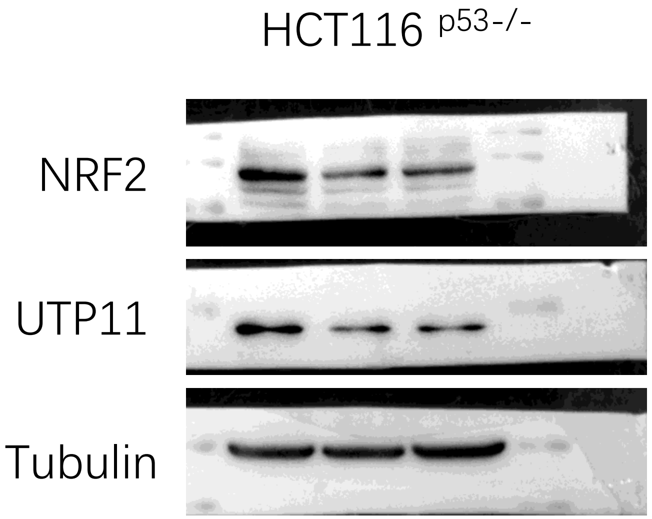

Figure 6P

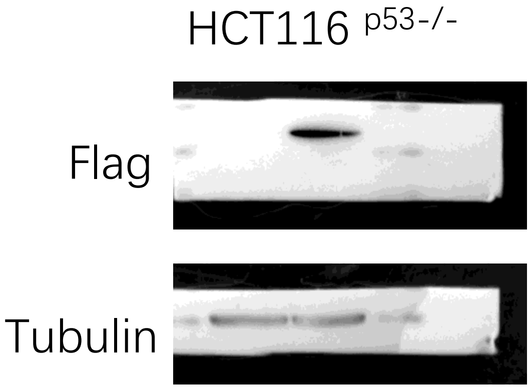

Figure 6U

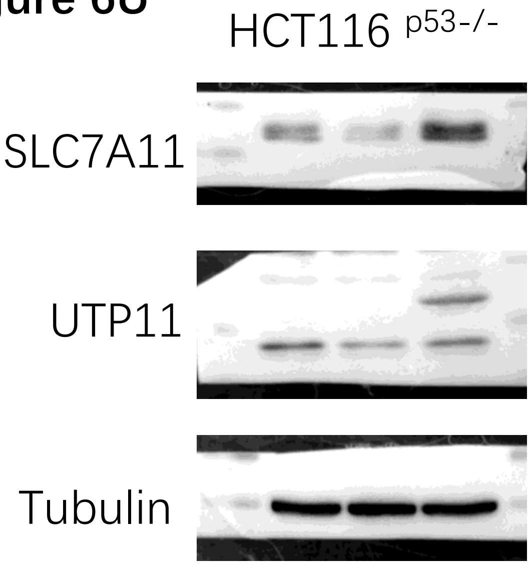

Figure 7A

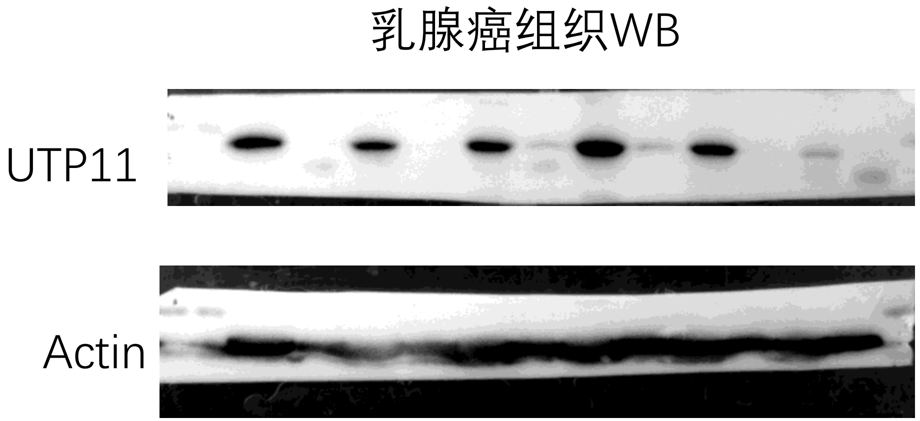

Supplement: Multimedia component 4 [file mmc4.pdf]
